# Clinical Relevance of Genetic Analysis in Patients With Pituitary Adenomas: A Systematic Review

**DOI:** 10.3389/fendo.2019.00837

**Published:** 2019-12-10

**Authors:** Medard F. M. van den Broek, Bernadette P. M. van Nesselrooij, Annemarie A. Verrijn Stuart, Rachel S. van Leeuwaarde, Gerlof D. Valk

**Affiliations:** ^1^Department of Endocrine Oncology, University Medical Center Utrecht, Utrecht, Netherlands; ^2^Department of Medical Genetics, Wilhelmina Children's Hospital, University Medical Center Utrecht, Utrecht, Netherlands; ^3^Department of Paediatrics, Wilhelmina Children's Hospital, University Medical Center Utrecht, Utrecht, Netherlands

**Keywords:** pituitary adenoma, germline mutation, genetic analysis, mutation, screening

## Abstract

Pituitary adenomas (PA) are amongst the most prevalent intracranial tumors, causing complications by hormonal overproduction or deficiency and tumor mass effects, with 95% of cases occurring sporadically. Associated germline mutations (*AIP, MEN1, CDKN1B, PRKAR1A, SDHx*) and *Xq26.3* microduplications are increasingly identified, but the clinical consequences in sporadic PA remain unclear. This systematic review evaluates predictors of a genetic cause of sporadic PA and the consequences for treatment outcome. We undertook a sensitive MEDLINE/Pubmed, EMBASE, and Web of Science search with critical appraisal of identified studies. Thirty-seven studies on predictors of mutations and 10 studies on the influence on treatment outcome were included. *AIP* and *MEN1* mutations were associated with young age of PA diagnosis. *AIP* mutations were also associated with gigantism and macroadenomas at time of diagnosis. *Xq26.3* microduplications were associated with PA below the age of five. *AIP* and *MEN1* mutation analysis is therefore recommended in young patients (≤30 years). *AIP* mutation analysis is specifically recommended for patients with PA induced gigantism and macroadenoma. Screening for *Xq26*.3 microduplications is advisable in children below the age of five with increased growth velocity due to PA. There is no evidence supporting mutation analysis of other genes in sporadic PA. *MEN1* mutation related prolactinoma respond well to dopamine agonists while *AIP* mutation associated somatotroph and lactotroph adenoma are frequently resistant to medical treatment. In patients harboring an *Xq26.3* microduplication treatment is challenging, although outcome is not different from other patients with PA induced gigantism. Effective use of genetic analysis may lead to early disease identification, while knowledge of the impact of germline mutations on susceptibility to various treatment modalities helps to determine therapeutic strategies, possibly lowering disease morbidity.

## Introduction

Pituitary adenomas (PAs) are amongst the most frequently encountered intracranial tumors with a reported prevalence for clinically relevant PAs of 68–98 per 100,000 (1–6). Pituitary adenomas are usually benign but can lead to clinical symptoms caused by hormonal overproduction or deficiency as well as by tumor mass. The majority of cases (95%) occur sporadically (7, 8). Familial clustering can be seen in the context of an inherited syndromic condition leading to an increased risk of PAs (most frequently Multiple Endocrine Neoplasia Type 1 (MEN1)) or without other (endocrine) manifestations in case of familial isolated pituitary adenoma (FIPA).

Clinical implications of identifying germline mutations in patients with PA, in terms of treatment and prognosis, have been reported by different authors (9–12). However, to our knowledge a complete overview of literature with thorough assessment of methodological quality of studies has not been performed to date. Detection of a germline mutation enables identifying family members at risk or occult disease burden in probands. Despite the clinical need, formal guidelines defining criteria for genetic screening of patients with apparently sporadic PA are scarce. In recent years, the amount of publications concerning germline mutations in (sporadic) pituitary adenoma has increased enormously. Despite all efforts, the mechanisms underlying pituitary tumorigenesis and the role of germline mutations in PAs in a sporadic setting remain poorly understood. Still, germline mutations are often not timely identified due to *de novo* mutations, low penetrance of hereditary syndromic conditions, unclear family history or small family size (13–15). The reported yield of genetic screening varies enormously, presumably due to a great variety of study populations, genetic screening methods and methodological quality of studies.

To provide a useful tool for daily practice in the frequently encountered dilemma whether or not to test for the presence of germline mutations in patients with apparently sporadic PA, we aim to determine the clinical value of genetic screening in apparently sporadic PA based on a rigorous systematic review and critical appraisal of the available literature.

## Methods

To assess the value of genetic testing in sporadic PA without syndromic features, we formulated two clinical questions for this review that are relevant for a physician when confronted with these patients: (1) what are predictors for the presence of a genetic cause of apparently sporadically occurring pituitary adenoma? (2) What is the impact of germline mutations on course of disease and treatment outcome of PA?

### Search Strategy and Study Selection

We performed a MEDLINE/Pubmed, EMBASE, and Web of Science search in November 2018. We applied a broad search strategy using “pituitary adenoma” and “genetic analysis” with an extensive list of synonyms. The complete search string is provided in Supplementary Data Sheet 1. We included human research written in English, French, German, or Dutch without restriction for year of publication. Publications using non-original data (reviews, letters to the editor, cohort duplicates) were only used for cross referencing, case-reports up to four cases were excluded.

Studies assessing predictors of a genetic cause of PA were included if (1) it was possible to retrieve data on sporadic cases separately and (2) (likely) pathogenic germline mutations of genes associated with PA were investigated. The genes of interest include the *MEN1, CDKN1B, CDKN2C, PRKAR1A, PRKACA, PRKACB, SDHx*, and *AIP* genes and microduplications of *Xq26.3*. Due to insufficient evidence in literature for *GPR101* allelic variants in the tumorigenesis of PA (15–21), studies on these variants were excluded from further review. Since the focus of this review is on patients with sporadically occurring PA, studies including patients with clear syndromic features suggestive for a certain genomic mutation were excluded.

Studies assessing the impact of a germline mutation on treatment outcome of PA were included if (1) results included information on treatment (type and number of treatments) and/or outcome (hormonal/disease control, tumor growth/reduction, complications) (2) information of the (sub)group of patients with a germline mutation was extractable and (3) at least five cases with a proven germline mutation were described.

After removal of duplications, two authors (MB and BN) independently screened all publications by title and abstract for possible relevance on the formulated questions. The full manuscript of all potentially eligible papers was then reviewed for in/exclusion by the same authors independently. In case of disagreement, consensus was reached by discussion, with the help of a third reviewer (RL). Reasons for exclusion at full text screening were recorded (See Supplementary Data Sheet 2). All included articles, reviews and case-reports were cross referenced for additional relevant articles.

### Data Extraction

Relevant data on study population (cohort origin, number of included patients, additional selection criteria, clinical subtype of adenoma, gender distribution, and familial status) and investigated gene(s) (including method(s) of genetic analysis and investigation(s) of pathogenicity) were extracted. The prevalence of the investigated germline mutations was obtained. Age, gender, adenoma size, and functionality were considered a potential predictor. Possible predictors of germline mutations were assessed if at least five cases with a germline mutation were identified in the study population. All quantitative data describing determinants of treatment outcome of PA in patients with proven germline mutations were extracted. In order to determine the predictive value of determinants and the effect on treatment outcome, a combination of effect size, statistical significance, reproducibility (number of studies with comparable results) and methodological quality of studies were taken into consideration.

### Critical Appraisal

For the systematic evaluation of risk of bias and applicability of studies on predictors of a genetic cause of PA, we adapted the Quality Assessment of Diagnostic Accuracy Studies tool (QUADAS-2) for our review purposes (22). For the evaluation of prognostic studies on the impact of germline mutation on treatment outcome, we customized the Quality In Prognosis Studies tool (QUIPS) (23). For more details, see Supplementary Data Sheets 3, 4. All included studies were appraised by two authors independently (MB and BN), in case of disagreement, consensus was reached by discussion or with the help of a third reviewer (RL). The strength of recommendations was graded using the Grading of Recommendations, Assessment, Development, and Evaluation system (24, 25).

## Results

### Study Selection

**Figure 1:**
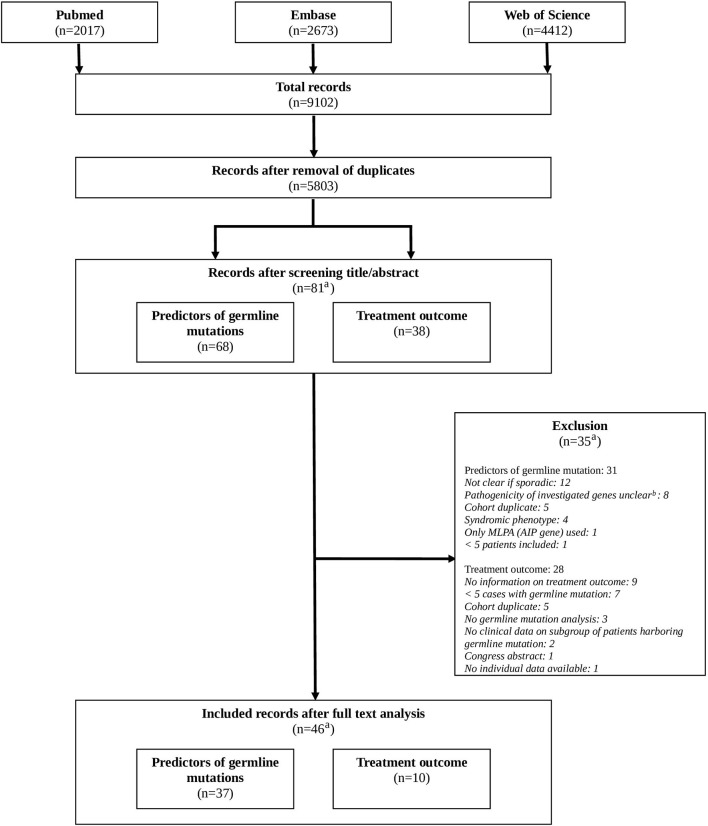
Flowchart. a: original records. Records can be included for both clinical questions (predictors of germline mutations, treatment outcome). b: *GPR101* allelic variants*, GNAI1/2/3, CABLES1, KCNQ1/2*, genome wide association studies, SNP allele frequencies studies.

After removal of duplicates a total of 5,803 original records were identified. After systematic screening, a total of 37 studies on possible predictors of germline mutations and 10 studies on the impact of a germline mutation on treatment outcome were included. One record was included for answering both clinical questions (26). Cross referencing did not result in additional relevant records. For further details, see Figure 1 (Flowchart).

### Predictors on Germline Mutation Status in Sporadic PA

Studies could be categorized into three separate groups: (i) patients with a somatotroph adenoma, (ii) young patients (≤30 years at diagnosis), and (iii) other groups of patients with PA.

#### Sporadic Somatotroph Adenoma

Out of 13 studies investigating the presence of an *AIP* gene mutation, one publication identified ≥ 5 cases with a germline mutation (27). In this study with a prevalence of an *AIP* mutation of 3.2%, predictors of the presence of a mutation were: younger age at diagnosis (mean age of *AIP* mutated patients 25 ± 10 vs. 43 ± 14 years in wildtype, *P* = 0.005) and gigantism (three out of five *AIP* mutated patients suffered from gigantism compared to 17 out of 149 patients without *AIP* mutation, *P* = 0.016). This study showed a minor risk of bias and intermediate applicability (see Tables 1A, 2A for more details).

**TABLE 1A:** Studies with sporadic somatotroph adenoma patients.

**References**	**Population**		**No. of sporadic patients^a^**	**Subtype adenoma**	**Investigated genes**	**Prevalence of mutations^b^**	**Possible predictors^c^**
	**Cohort**	**Additional selection criteria**					
Yamasaki et al. (28)	Japan	GH or GH/PRL secreting PA	32	GH = 30 GH/PRL = 2	*PRKAR1A*	0	N/A
Vierimaa et al. (29)	Finland	Acromegalic patients	10^d^	GH = 10	*AIP*	20% (2 pt)	N/A (2 cases)
Cazabat et al. (27)	France	GH-secreting PA Exclusion of patients with clinical features suggesting MEN1, CNC, or MAS	154	GH = 154	*AIP, MEN1, PRKAR1A*^e^	*AIP*: 3.2% (5 pt)^f^	Younger age Gigantism *Male gender*
Iwata et al. (30)	Japan	GH secreting PA	40	GH = 40	*AIP*	2.5% (1 pt)	N/A (1 case)
Georgitsi et al. (31)	Finland	Acromegaly	50	GH = 50	*CDKN1B*	0	N/A
Leontiou et al. (32)	International^g^	Acromegalic patients^g^	37	GH = 37	*AIP*	0	N/A
Occhi et al. (33)	Italy	Acromegaly	131	GH = 131	*AIP, CDKN1B*^h^	*AIP*: 3.1% (4 pt) *CDKN1B*: 0	N/A (4 cases)
Oriola et al. (34)	Spain	GH secreting PA Resistant to SSA	50	GH = 50	*AIP*	4% (2 pt)	N/A (2 cases)
Zatelli et al. (35)	Italy	GH secreting	16	GH = 16	*AIP*	0	N/A
Trivellin et al. (16)	International	Xq26.3 duplication: gigantism	38	GH = 38	*Xq26.3 duplication*^i^	*Xq26.3*: 24% (9 pt)	N/A (insufficient data)^i^
Schöfl et al. (36)^j^	Germany	Acromegaly Age at diagnosis <30 years	87	GH = 87	*AIP*	2.3% (2 pt)	N/A (2 cases)
Karaca et al. (37)	Turkey	GH producing PA	92	GH = 92	*AIP*	1.1% (1 pt)	N/A (1 case)
Ferrau et al. (19)	Italy	GH producing PA	210	GH = 210	*AIP*	*AIP*: 1.9% (4 pt)	N/A (4 cases)
Mangupli et al. (38)	Venezuela	Pituitary gigantism	8	GH = 8	*AIP, MEN1, Xq26.3 duplication*^k^	*AIP*: 43% (3 pt) Xq26.3: 0 *MEN1*: ?^k^	N/A (3 cases)
Matsumoto et al. (39)	Japan	GH producing PA	61^l^	GH = 61	*AIP*	4.9% (3 pt)	N/A (3 cases)
Ozkaya et al. (40)	Turkey	GH producing PA Exclusion of patients with preoperative SRL treatment or poor adherence Exclusion of MEN1, CNC, MAS	94^m^	GH = 94	*AIP*	2.1% (2 pt)	N/A (2 cases)

*CNC, Carney complex; MAS, McCune-Albright Syndrome; MEN1, multiple endocrine neoplasia type 1; N/A, not applicable; PA, pituitary adenoma; pt, patients; SSA, somatostatin analogs*.

*GH, somatotroph adenoma; PRL, lactotroph adenoma; ACTH, corticotroph adenoma; TSH, thyrotroph adenoma; NFPA, non-functioning pituitary adenoma; GH/PRL, mixed somatotroph/lactotroph adenoma*.

*Cursive predictors: suggestive predictor but no statistical significance reached/insufficient data to calculate statistical significance*.

a *Only (groups of) patients are included of which the sporadic status could be determined*.

b *Mutations or variants that were considered pathogenic or likely pathogenic by the authors of each study*.

c *Possible predictors are presented if a minimum of five cases of patients with a germline mutation are reported*.

d *Possible founder effect (frequent occurrence of the Q14X mutation in the Finnish cohort)*.

e *MEN1 and PRKAR1A gene analysis was performed in patients <30 years of age without AIP mutation (n = 28)*.

f *Another patient harbored a missense R304Q (c.911G >A) p.Arg304Gln mutation with conflicting interpretations of pathogenicity. Many later publications, e.g. Occhi et al. (33), Tichomirowa et al. (26), Cuny et al. (13), Preda et al. (41), Tuncer et al. (42) considered this a (likely) pathogenic variant*.

g *Seven cases of childhood-onset gigantism (Australia, UK, Brazil, USA), 30 adult-onset acromegaly cases (cohort unknown)*.

h *CDKN1B gene analysis was performed in a subgroup of 38 patients with multiple tumors*.

i *There is insufficient information provided to determine possible predictors*.

j *This study is also presented in Table 1B*.

k *AIP and MEN1 gene analysis was performed in seven patients. The results of MEN1 gene analysis are not reported*.

l *It cannot be excluded that a part of this study population is previously reported in Iwata et al. (30)*.

m *It cannot be excluded that a part of this study population is previously reported in Yarman et al. (47) and/or Tuncer et al. (42)*.

**TABLE 1B:** Studies with young (≤30 years) sporadic pituitary adenoma patients.

**References**	**Population**		**No. of sporadic patients^a^**	**Subtype adenoma**	**Investigated genes**	**Prevalence of mutations^b^**	**Possible predictors^c^**
	**Cohort**	**Additional selection criteria**					
Georgitsi et al. (43)	Italy	Age at disease onset or diagnosis <18 years Exclusion of family history of MEN1	36^d^	GH = 5 PRL = 19 ACTH = 3 NFPA = 7^e^ GH/PRL = 2	*AIP*	2.8% (1 pt)	N/A (1 case)
Stratakis et al. (44)	USA (Bethesda)	Age at diagnosis ≤18 years AND (1) Cushing disease or (2) GH/PRL secreting PA	80	GH = 3 PRL = 3 ACTH = 74	*AIP, MEN1, CDKN1B, CDKN2C, PRKAR1A*	*AIP*: 3.8% (3 pt) *MEN1*: 1.3% (1 pt)	N/A (4 cases)
Tichomirowa et al. (26)	International^f^	Age at diagnosis <30 years Macroadenoma (≥10 mm on MRI)	163	GH = 83 PRL = 61 ACTH = 2 TSH = 1 NFPA = 16^g^	*AIP*	11.7% (19 pt)	Younger age *Extrasellar extension* *Male gender*
Cuny et al. (13)	France	Age at diagnosis <30 years Macroadenoma (≥10 mm on MRI) Exclusion of patients with hypercalcemia	174^h^	GH = 79 PRL = 74 ACTH = 8 TSH = 1 NFPA = 12^i^	*AIP, MEN1*	*AIP*: 8.6% (15 pt) *MEN1*: 3.4% (6 pt)	*Younger age* *(AIP & MEN1)* *Extrasellar extension (AIP)* *Gigantism (AIP)* *Male gender* (*AIP*) *NFPA (AIP)* *Prolactinoma (MEN1)*
Schöfl et al. (36)^j^	Germany	Acromegaly Age at diagnosis <30 years	87	GH = 87	*AIP*	2.3% (2 pt)	N/A (2 cases)
Hernandez-Ramirez et al. (45)	International	Age at disease onset ≤30 years	404^k^	GH = 290 PRL = 67 ACTH = 21 TSH = 2 NFPA = 21^l^ Other = 3^m^	*AIP, MEN1, CDKN1B*^n^	*AIP*: 8.4% (34 pt) *MEN1*: 0 *CDKN1B*: 0	Younger age Macroadenoma Extrasellar extension *Gigantism* *GH secreting PA*

*N/A, not applicable; MEN1, multiple endocrine neoplasia type 1; PA, pituitary adenoma; pt, patients*.

*GH, somatotroph adenoma; PRL, lactotroph adenoma; ACTH, corticotroph adenoma; TSH, thyrotroph adenoma; NFPA, non-functioning pituitary adenoma; GH/PRL, mixed somatotroph/lactotroph adenoma*.

*Cursive predictors: suggestive predictor but no statistical significance reached/insufficient data to calculate statistical significance*.

a *Only (groups of) patients are included of which the sporadic status could be determined*.

b *Mutations or variants that were considered pathogenic or likely pathogenic by the authors of each study*.

c *Possible predictors are presented if a minimum of five cases of patients with a germline mutation are reported*.

d *Three patients previously reported in Georgitsi et al. (46)*.

e *Adenoma subtype is based on “clinical diagnosis”*.

f *Belgium, Brazil, Bulgaria, Czech Republic, France, Germany, Italy, Lebanon, and Spain*.

g *Definition of NFPA is not provided. The tumor of the only NFPA patient with a germline AIP mutation was negative for all pituitary hormones on immunohistochemistry*.

h *Fifty-nine patients previously reported in Tichomirowa et al. (26)*.

i *Definition of NFPA is not provided. The tumor of one NFPA patient with a germline AIP mutation had a partial (50%) immunoreactivity for GH without any pituitary hormonal hypersecretion in vivo (silent somatotroph adenoma). The tumors of the other three NFPA patients with a germline AIP or MEN1 mutation were non-reactive on immunostaining experiments*.

j *This study is also presented in Table 1A*.

k *Six patients previously reported in Leontiou et al. (32)*.

l *Definition of NFPA is not provided. Immunohistochemistry results were available in 103 (out of 404) patients. All sporadic patients with a germline AIP mutation and available histopathology results (n = 14) had GH positive pituitary adenomas by immunohistochemistry. In the group of sporadic patients with available histopathology results but without germline AIP mutation (n = 89), three tumors were non-reactive (null cell PA)*.

m *One FSH-secreting PA, two not specified*.

n *MEN1 gene analysis is performed in 33 patients, CDKN1B gene analysis is performed in one patient*.

**TABLE 1C:** Studies with other groups of sporadic pituitary adenoma patients.

**References**	**Population**		**No. of sporadic patients^a^**	**Subtype adenoma**	**Investigated genes**	**Prevalence of mutations^b^**	**Possible predictors^c^**
	**Cohort**	**Additional selection criteria**					
Zhuang et al. (56)	USA (Bethesda), Canada (Toronto)	Patients who had undergone full preoperative endocrine evaluation	38	GH = 8 PRL = 8 ACTH = 14 TSH = 1 Other = 7^d^	*MEN1*	0	N/A
Schmidt et al. (57)	Germany	Exclusion of patients with a familial history of MEN1-associated tumors	61	GH = 16 PRL = 6 ACTH = 1 TSH = 1 NFPA = 37^e^	*MEN1*	0	N/A
Farrell et al. (58)	UK	Patients previously shown to harbor allelic deletion on 11q13	23	GH = 15 PRL = 2 ACTH = 1 NFPA = 5^f^	*MEN1*	0	N/A
Yu et al. (50)	USA (Los Angeles)	–	63	GH = 35 PRL = 15 ACTH = 5 NFPA = 8^g^	*AIP*	0	N/A
DiGiovanni et al. (51)	Canada	–	66	GH = 50 Other = 16^h^	*AIP*	0	N/A
Barlier et al. (52)	France, Belgium, Italy	Exclusion of a history of MEN1 or CNC	107	GH = 26 PRL = 49 ACTH = 2 TSH = 1 NFPA = 29^i^	*AIP*	0	N/A
Georgitsi et al. (46)	USA (Cleveland), Italy	USA (n = 113): patients undergoing PA resectionItaly (n = 71): acromegaly	184	GH = 84 PRL = 11 ACTH = 13 NFPA = 76^j^	*AIP*	1.1% (2 pt)	N/A (2 cases)
Buchbinder et al. (54)	Germany	Exclusion of MEN1 en CNC	110	GH = 10 PRL = 38 ACTH = 5 NFPA = 55^k^ Other = 2^l^	*AIP*	2.7% (3 pt)^m^	N/A (3 cases)
Cai et al. (55)	China	–	216	GH = 80 PRL = 39 ACTH = 39 NFPA = 58^n^	*AIP*	2.8% (6 pt)	*Younger age* *GH secreting PA Male gender*
Preda et al. (41)	UK	Adult patients with age at disease onset ≤40 years	127	GH = 48 PRL = 43 ACTH = 15 TSH = 1 NFPA = 20^o^	*AIP*	1.6% (2 pt)	N/A (2 cases)
Yarman et al. (47)	Turkey	Functional PA	91	GH = 47 PRL = 21 ACTH = 23	*AIP*	0	N/A
Lecoq et al. (15)	France	–	766^p^	GH = 218 PRL = 256 ACTH = 68 TSH = 14 NFPA = 165^q^ GH/PRL = 45	*AIP*	*AIP*: 2.9% (22 pt)	N/A (insufficient data)^r^
De Sousa et al. (48)	Australia	Age of onset ≤40 years^s^	30	?	*AIP, MEN1, CDKN1B, PRKAR1A and SDHx*^t^	*AIP*: 13.3% (4 pt)Other genes: 0	N/A (4 cases)
Araujo et al. (49)	Brazil	Macroadenoma diagnosed * ≤* 40 years or adenoma of any size diagnosed < 18 years of age	132	GH = 74 PRL = 38 ACTH = 10 NFPA = 10^u^	*AIP*	2.3% (3 pt)	N/A (3 cases)
Foltran et al. (53)	Brazil	GH producing PA or NFPA	62	GH = 41 NFPA = 21^v^	*AIP*	0	N/A
Tuncer et al. (42)	Turkey	Functional PA No clinical suggestions of MEN1 or CNC Age at diagnosis ≤ 40 years for PRL and ACTH producing PA, age at symptom onset ≤ 40 years for GH producing PA	97^w^	GH = 55 PRL = 25 ACTH = 17	*AIP*	2.1% (2 pt)	N/A (2 cases)

*CNC, Carney complex; MEN1, multiple endocrine neoplasia type 1; N/A, not applicable; PA, pituitary adenoma, pt, patients*.

*GH, somatotroph adenoma; PRL, lactotroph adenoma; ACTH, corticotroph adenoma; TSH, thyrotroph adenoma; NFPA, non-functioning pituitary adenoma; GH/PRL, mixed somatotroph/lactotroph adenoma*.

*Cursive predictors: suggestive predictor but no statistical significance reached/insufficient data to calculate statistical significance*.

a *Only (groups of) patients are included of which the sporadic and non-syndromic status could be determined*.

b *Mutations or variants that were considered pathogenic or likely pathogenic by the authors of each study*.

c *Possible predictors are presented if a minimum of five cases of patients with a germline mutation are reported*.

d *Two oncocytomas, two mixed (GH/PRL) PAs, one gonadotroph PA, one glycoprotein PA, one PRL+ ACTH PA (two separate PA with independent biochemical function)*.

e *Subtype definition based on pre-operative hormonal status*.

f *Definition of NFPA is not provided*.

g *“Clinically non-functioning adenomas,” without further specification*.

h *No further subtype specification or subtype definitions*.

i *Definition of NFPA is not provided*.

j *Definition of NFPA is not provided. All patients from the USA cohort (n = 113) underwent biochemical and immunohistochemistry confirmed diagnosis*.

k *An adenoma was declared as non-functioning when it was associated with levels of TSH, ACTH, PRL, and GH in the normal range*.

l *Two gonadotropinomas*.

m *Two of these patients harbored a R16H (c.47G > A) mutation, which other authors (Georgitsi et al. (46), Cazabat et al. (27), Ferrau et al. (19)) considered not (likely) pathogenic*.

n *Definition of NFPA is not provided*.

o *Definition of NFPA is not provided*.

p *This cohort includes all 443 patients reported in Cazabat et al. (59) (which is therefore excluded from this review)*.

q *Including both NFPA and gonadotropinomas. Adenoma subtype was based on clinical, biological and/or histological criteria*.

r *No information on subgroup of only (likely) pathogenic mutations are presented*.

s *Other subgroups of patients (family and/or personal history of endocrine neoplasia) are excluded*.

t *SDHA, SDHB, SDHC, SDHD*.

u *Definition of NFPA is not provided. Immunohistochemical staining was performed in cases who underwent surgery*.

v *Definition of NFPA is not provided. Tumor samples for immunohistochemical staining were available in 45 out of 62 cases (NFPA: 18 out of 21 cases)*.

w *Fifty-six patients previously reported in Yarman et al. (47)*.

**TABLE 2A:** Studies with sporadic somatotroph adenoma patients.

**References**	**Gene(s) studied**	**Risk of bias**			**Applicability**	
		**Patient selection**	**Reference standard**	**Flow and timing**	**Patient selection**	**Reference standard**
Yamasaki et al. (28)	*PRKAR1A*	–	±	±	±	–
Vierimaa et al. (29)	*AIP*	–	–	±	–	–
Cazabat et al. (27)	*AIP, MEN1, PRKAR1A*	+	+	±	±	±
Iwata et al. (30)	*AIP*	–	–	±	±	-
Georgitsi et al. (31)	*CDKN1B*	–	+	+ +	-	±
Leontiou et al. (32)	*AIP*	–	+	++	±	±
Occhi et al. (33)	*AIP, CDKN1B*	–	*+ +*	±	±	+
Oriola et al. (34)	*AIP*	–	+ +	+ +	±	+
Zatelli et al. (35)	*AIP*	–	+	+ +	–	±
Trivellin et al. (16)	*Xq26.3 duplication*	–	+ +	–	–	+
Schöfl et al. (36)^a^	*AIP*	+ +	+ +	+ +	±	+
Karaca et al. (37)	*AIP*	–	+	+ +	±	±
Ferrau et al. (19)	*AIP*	+	– –	+ +	±	–
Mangupli et al. (38)	*AIP, MEN1, Xq26.3 duplication*	+	+	– –	±	–
Matsumoto et al. (39)	*AIP*	+	+	–	±	+
Ozkaya et al. (40)	*AIP*	–	+	+ +	±	±

a *This study is also presented in **Table 2B***.

In only two studies on *Xq26.3* microduplication the data of apparently sporadically occurring PA could be extracted (16, 38). Both were at risk of bias and had a relatively low applicability for daily clinical practice. Trivellin et al. found an *Xq26.3* duplication in 9 out of 38 sporadic patients with pituitary gigantism (24%). The total group of germline affected patients with gigantism (14 out of 43) had a female predominance (71 vs. 24%, *P* = 0.007), much earlier onset of increased growth velocity (median age 1.0 year (range 0.5–2.0) vs. 16.0 year (range 5.0–18.0), *P* < 0.001) and higher insulin-like growth factor (IGF-1) levels and more frequently elevated prolactin levels at diagnosis. Mangupli et al. found no cases of *Xq26.3* microduplication at all.

In the five studies investigating the presence of *MEN1, CDKN1B*, and/or *PRKAR1A* mutations in sporadically occurring somatotroph adenoma, no predictors were identified (27, 28, 31, 33, 38).

The outcomes of all included studies on sporadic somatotroph adenoma are presented in Table 1A. Methodological quality assessment of studies is presented in Table 2A. For further details on study results, see Supplementary Data Sheet 5.

#### Young (≤30 Years) Patients With Sporadic PA

Three studies assessing the presence of an *AIP* mutation identified ≥5 cases with a germline mutation, reporting a mutation prevalence of 8.4, 8.6, and 11.7%, respectively (13, 26, 45). Study characteristics of all studies are displayed in Table 1B.

In all studies, the presence of an *AIP* mutation was related with a younger age of onset or, inversely, prevalence of *AIP* mutations was higher in patients with a younger age of diagnosis (≤18 years). Furthermore, the two studies only including patients with macroadenoma (≥10 mm) reported the highest frequency of *AIP* mutations, illustrating that macroadenoma is a predictor of this specific mutation. Extrasellar extension was a frequent feature. Thirdly, *AIP* mutations were more likely identified in patients suffering from gigantism. Additionally, despite a nearly equal gender distribution in study populations, male gender was overrepresented in *AIP* mutated patients.

Data on adenoma subtype were conflicting: although Cuny et al. reported a higher prevalence of *AIP* mutation in non-functioning PA, results from Hernandez-Ramirez et al. showed all *AIP* mutation related PA to be somatotroph adenomas. For further details on study results, see Supplementary Data Sheet 5.

The study of Cuny et al. showed only minor risk of bias and good applicability, making these results more reliable. Full quality assessment of studies can be found in Table 2B.

**TABLE 2B:** Studies with young (≤30 years) sporadic pituitary adenoma patients.

**References**	**Gene(s) studied**	**Risk of bias**			**Applicability**	
		**Patient selection**	**Reference standard**	**Flow and timing**	**Patient selection**	**Reference standard**
Georgitsi et al. (43)	*AIP*	+	+	+ +	±	±
Stratakis et al. (44)	*AIP, MEN1, CDKN1B^a^, PRKAR1A*	–	+	+ +	–	±
Tichomirowa et al. (26)	*AIP*	–	+ +	+ +	±	+
Cuny et al. (13)	*AIP, MEN1*	+	+ +	+ +	±	+
Schöfl et al. (36)^b^	*AIP*	+ +	+ +	+ +	±	+
Hernandez-Ramirez et al. (45)	*AIP, MEN1, CDKN1B*	–	+ +	±	–	+

a *CDKN2C was also investigated*.

b *This study is also presented in **Table 2A***.

Regarding MEN 1 mutations, the study of Cuny et al. was at the lowest risk of bias and highest applicability (13). In this series of patients younger than 30 years (prevalence of *MEN1* mutation: 3.4%), patients with a *MEN1* mutation tended to be younger: 3 out of 46 (6.5%) patients ≤ 18 years harbored a germline *MEN1* mutation vs. 3 out of 128 (2.3%) patients from 19 to 30 years at diagnosis. *MEN1* mutations did also occur more frequently in prolactinomas (5.4%) than other PA subtypes (2%).

In the studies on the presence of the *CDKN1B, CDKN2C*, and *PRKAR1A* gene mutations no germline mutations were identified (44, 45).

#### Other Groups of Patients With Sporadic PA

Sixteen studies applied a different set of in- and exclusion criteria than somatotroph adenoma or age at diagnosis ≤30 years, although four publications did use age criteria (41, 42, 48, 49). The reported prevalence of germline mutations within these studies is relatively low, with the exception of one study reporting a prevalence of 13.3 % (48).

The presence of *AIP* mutations was assessed in 13 studies. No *AIP* mutation was found in five of these studies (47, 50–53) and six studies described one to four cases with *AIP* mutation (41, 42, 46, 48, 49, 54). Lecoq et al. detected 22 cases, but unfortunately there was insufficient data reported for the identification of possible predictors of *AIP* status (15). In a publication of high methodological quality, Cai et al. detected six persons with *AIP* mutations (2.8%) in a group of 216 Han Chinese sporadic PA patients (55). The prevalence of an *AIP* mutation was higher in patients with a younger age at diagnosis (patients ≥ 18 years 6.3 vs. 2.5% in patients ≥ 18 years at diagnosis) and in the subgroup of somatotroph adenoma (6.3 vs. 0.7% in non-GH producing PA). In this study, male gender also appeared to be related with a higher prevalence of *AIP* mutations (5.3 vs. 0.8%).

Four studies on predictors for *MEN1* gene mutations (48, 56–58) and one study on *CDKN1B, PRKAR1A*, and *SDHx* (48) did not reveal any mutation in the patients under study. See Table 1C for further study detail and Table 2C for all results on quality assessment.

**TABLE 2C:** Studies with other groups of sporadic pituitary adenoma patients.

**References**	**Gene(s) studied**	**Risk of bias**			**Applicability**	
		**Patient selection**	**Reference standard**	**Flow and timing**	**Patient selection**	**Reference standard**
Zhuang et al. (56)	*MEN1*	–	– –	±	+	–
Schmidt et al. (57)	*MEN1*	+	– –	+ +	–	–
Farrell et al. (58)	*MEN1*	– –	–	++	–	–
Yu et al. (50)	*AIP*	–	– –	+ +	+	–
DiGiovanni et al. (51)	*AIP*	–	– –	±	–	–
Barlier et al. (52)	*AIP*	– –	+ +	+ +	±	+
Georgitsi et al. (46)	*AIP*	–	+	±	–	±
Buchbinder et al. (54)	*AIP*	–	+	+ +	+	±
Cai et al. (55)	*AIP*	+	+ +	+ +	+	+
Preda et al. (41)	*AIP*	+	+ +	+ +	±	+
Yarman et al. (47)	*AIP*	–	–	+ +	±	±
Lecoq et al. (15)	*AIP*	+	+ +	±	+	+
De Sousa et al. (48)	*AIP, MEN1, CDKN1B, PRKAR1A, SDHx*	–	+ +	±	–	+
Araujo et al. (49)	*AIP*	+	+ +	±	±	+
Foltran et al. (53)	*AIP*	–	+	+ +	±	±
Tuncer et al. (42)	*AIP*	–	+ +	+ +	–	+

### Impact of a Germline Mutation on Treatment Outcome in PA

Ten studies reported on treatment outcome in patients with a germline mutation. In seven publications, treatment outcome was compared with a cohort of patients without germline mutation. Study characteristics are presented in Table 3.

**TABLE 3:** Study characteristics of studies assessing the impact of a germline mutation on treatment outcome.

**References**	**Population**			**No. of patients with germline mutation^a^**	**No. of patients without germline mutation**	**Subtype adenoma** ** (germline/** ** wildtype)**	**Investigated treatment outcome**
	**Cohort**	**Gene(s)**	**Additional selection criteria**				
Verges et al. (60)	Belgium, France	*MEN1*	MEN1 based on clinical or genetic criteria Non-MEN1 PA were matched for age, year of diagnosis, and FU period	136	110	GH = 12/15 PRL = 85/68 ACTH = 6/7 NFPA = 20/18^b^ Mixed = 13/2	Normalization of hypersecretion
Daly et al. (9)	International^c^	*AIP*	GH producing PA	75^d^	232	GH = 75/232	Treatment characteristics, controlled and active disease, hypopituitarism
Tichomirowa et al. (26)	International^e^	*AIP*	Sporadic Age at diagnosis <30 years Macroadenoma (≥10 mm on MRI)	19	144^f^	GH = 11/72 PRL = 7/54 ACTH = 2 TSH = 1 NFPA = 1/15^g^	Treatment characteristics, disease control, tumor shrinkage
Beckers et al. (61)	International	*Xq26.3*	Xq26.3 duplication: gigantism	18^h^	–^f^	GH = 18	Treatments characteristics, hormonal control, tumor shrinkage, hypopituitarism
De Laat et al. (62)	Netherlands	*MEN1*	MEN1: based on clinical or genetic criteria ≥ 16 years of age	123	–^f^	GH = 8 PRL = 52 ACTH = 4 NFPA = 52^i^ Mixed = 5 Other = 2^j^	Tumor growth, control of excess hormonal secretion
Salenave et al. (63)	France	*AIP* *MEN1*	Macroprolactinoma < 20 years of age	*AIP*: 5 *MEN1*: 3	AIP: 50^k^ MEN1:59^k^	PRL = 59	DA resistance
Rostomyan et al. (14)	International^l^	*AIP* *Xq26.3*	Gigantism	*AIP*: 42 *Xq26.3*: 14	77^m^	GH = 42/14/77	Multimodal treatment, GH/IGF-1 control at FU, age when control achieved, long-term control, hypopituitarism
Iacovazzo et al. (17)	International^n^	*AIP* *Xq26.3*	Gigantism and acromegaly patients Exclusion of MEN1, CNC, MAS	*AIP*: 63 *Xq26.3*: 12	78	GH = 63/12/78	Number of treatments, hypopituitarism
Nagata et al. (64)	Japan	*AIP* *PRKAR1A*	GH producing PA Age of diagnosis ≤ 20 years	*AIP*: 5 *PRKAR1A*: 2	18^o^	GH = 5/2/18	Hormonal control
Caimari et al. (65)	International^p^	*AIP*	FIPA or age at disease onset ≤ 30 years or referred patients Exclusion of MEN1, MEN4, CNC, X-LAG, DICER1 syndrome	134	1271	GH = 119/648^q^ PRL = 11/333 ACTH = 0/74 NFPA = 4/181^r^ Other = 0/11^s^	Number of treatments

*CNC, Carney complex; DA, dopamine agonist; FIPA, familial isolated pituitary adenoma; FU, follow-up; GH, growth hormone; IGF-1, insulin-like growth factor 1; MAS, McCune-Albright Syndrome; MEN1, multiple endocrine neoplasia type 1; MEN4, multiple endocrine neoplasia type 4; PA, pituitary adenoma; X-LAG, X-linked Acrogigantism*.

*GH, somatotroph adenoma; PRL, lactotroph adenoma; ACTH, corticotroph adenoma; TSH, TSH secreting adenoma; NFPA, non-functioning pituitary adenoma*.

a *Mutations or variants that were considered pathogenic or likely pathogenic by the authors of each study*.

b *Diagnosis of adenoma subtype was made based on (increased) plasma levels of pituary hormones. Immunohistochemistry data were available in 42 cases. In 2 out of 15 cases of NFPA histologically examined, immunostaining was positive for LH and FSH*.

c *Belgium, Finland, France, Italy, Spain, Germany, Bulgaria, Netherlands, Brazil, Argentina, the United States of America, Australia, New Zealand, and Lebanon*.

d *The study included 96 patients with AIP mutation (of which 41 reported for the first time). The clinical behavior of somatotropinoma adenoma (n = 75) is compared with controls*.

e *Belgium, Brazil, Bulgaria, Czech Republic, France, Germany, Italy, Lebanon, and Spain*.

f *No comparison is made with wildtype PA*.

g *Definition of NFPA is not provided. The immunohistochemical staining of the tumor of the only NFPA patient with a germline AIP mutation was negative*.

h *Thirteen patients previously reported in Trivellin et al. (16)*.

i *Adenoma subtype classification was based on laboratory test results, no immunohistochemistry data available*.

j *Two gonadotroph adenomas*.

k *The study included 77 patients with macroprolactinoma. Germline mutation analysis was conducted in 50 patients (AIP) and 59 patients (MEN1)*.

l *Argentina, Australia, Belgium, Brazil, Bulgaria, Canada, Denmark, India, Italy, Finland, France, Germany, New Zealand, Romania, Russia, Spain, the Netherlands, and the United States of America*.

m *The study included 208 patients with pituitary gigantism. In 143 patients genetic analysis was performed. Seven cases of MAS, two cases of CNC and one case of MEN1 were excluded from this comparison*.

n *Not further specified. it cannot be excluded that a part of this study population is previously reported. One X-LAG patient was previously described in Trivellin et al. (16), one X-LAG patient was previously described in Beckers et al. (61)*.

o *The study included 25 patients. Only 13 patients were tested for AIP mutations. Negative germline analysis and no germline analysis is reported as “no mutation” in this study*.

p *Not further specified. it cannot be excluded that a part of this study population is previously reported*.

q *Including PA with prolactin cosecretion*.

r *Definition of NFPA is not provided*.

s *Any other type of functioning pituitary tumor*.

All seven studies on *AIP* mutations showed a potential risk of (patient) selection bias. The study of Daly et al. was at lowest risk of bias (9) (see Table 4 for full reporting of quality assessment). In this study 75 patients with an *AIP* mutation associated somatotroph adenoma were compared with 232 somatotropinomas without an *AIP* mutation. The proportion of patients receiving multimodal treatment was comparable (61.3 vs. 66.4%, respectively) and there was no significant difference in disease control (70.4 vs. 80.5%, respectively, *P* = 0.06). There were however some clear discrepancies in treatment characteristics and outcome: among patients with a higher cumulative treatment burden (≥3 distinct modalities), long-term disease control rates were significantly worse in *AIP* mutation associated adenoma (55.6 vs. 82.9%, *P* = 0.01). Furthermore, somatostatin analog (SSA)-induced GH and IGF-1 reduction and tumor size reduction was significantly less in *AIP* mutation associated PA. In line with these data, patients harboring an *AIP* mutation more often underwent a reoperation (21.9 vs. 5.5%). Although the prevalence of hypopituitarism in follow-up did not differ (*AIP* mutation associated 22.5 vs. controls 25.2%), patients with *AIP* mutation had a significantly higher number of pituitary deficiencies. Other studies on *AIP* mutation associated somatotropinomas showed similar results (26, 64). One study focused on *AIP* mutations in patients with apparently sporadically occurring PA and not familial cases (26). In this study, 4 out of 11 (36%) patients with *AIP* mutations underwent multiple surgical interventions, while post-operative SA therapy achieved disease control in only one out of nine patients.

**TABLE 4:** Quality assessment of studies assessing the impact of a germline mutation on treatment outcome.

**References**	**Gene(s) studied**	**Risk of bias**			
		**Patient selection**	**Determination of germline status**	**Outcome measurement**	**Analysis and reporting**
Verges et al. (60)	*MEN1*	+ +	– –	–	+ +
Daly et al. (9)	*AIP*	±	+ +	+ +	+ +
Tichomirowa et al. (26)	*AIP*	–	+ +	– –	±
Beckers et al. (61)	*Xq26.3*	±	+ +	–	±
De Laat et al. (62)	*MEN1*	*+ +*	±	*+ +*	±
Salenave et al. (63)	*AIP, MEN1*	±	– –	±	±
Rostomyan et al. (14)	*AIP, Xq26.3*	+	– –	–	+ +
Iacovazzo et al. (17)	*AIP, Xq26.3*	–	– –	– –	+ +
Nagata et al. (64)	*AIP, PRKAR1A*	±	– –	+ +	±
Caimari et al. (65)	*AIP*	±	+ +	–	+ +

Two studies focused on patients with PA induced gigantism. Since these patients represent a distinct group with particularly high disease severity, these results are separately displayed. In contrast, Rostomyan et al. reported better treatment outcomes in *AIP* mutation associated gigantism than in patients suffering from gigantism without genetic abnormalities (14). Within an international cohort of 208 patients with pituitary gigantism, hormonal control was more frequently reached in *AIP* mutation associated PA. Multimodal treatment was seldom necessary in *AIP* mutation associated somatotropinoma gigantism (23.8 vs. 42.7% in controls, *P* = 0.04). Long-term control (>12 months) was reached more often in the *AIP* mutated patients (55.3 vs. 38.4%), but this was not statistically significant (*P* = 0.08). The frequency of hypopituitarism at follow-up was similar between both groups (73 vs. 66%). In another study including 153 patients with PA induced gigantism, no significant difference in number of treatments or in prevalence of hypopituitarism was found between 63 patients with *AIP* mutation associated gigantism and patients with gigantism but without genetic abnormalities (17).

In search for factors associated with response to dopamine agonists in macroprolactinoma, Salenave et al. found *AIP* mutations not to be a significant determinant. However, in this study only a small sample of *AIP* mutated PA (*n* = 4) was included (63). Failure of dopamine agonists in *AIP* mutation related PA has been described frequently (50% of cases) in other studies as well and multiple surgical interventions were needed regularly (9, 26). In the cohort of *AIP* mutations in apparently sporadically occurring PA (26), five out of seven patients (71%) underwent surgery and four out of seven patients (66.7%) had to undergo multiple surgeries, which was comparable with results from another study cohort of mainly familial *AIP* cases (9).

No comparative data have been published on treatment outcome in *AIP* mutation associated vs. wildtype non-functioning PA (NFPA). However, Daly et al. did report seven cases with *AIP* mutation related NFPA: six patients underwent surgery (of which one also underwent radiotherapy), long-term control of tumor size was achieved in all cases (9).

One of the largest studies on *AIP* mutation associated PA (134 cases) showed a trend toward a higher number of treatments in both functioning and non-functioning *AIP* mutation related PA (median 2 (IQR 1–3)) compared to patients without mutation (*n* = 1,271, median 1 (IQR 1–2)) (*P* = 0.055) (65). All data are shown in Supplementary Data Sheet 5.

Treatment-related outcome of PAs in MEN1 patients was described in three studies (60, 62, 63). A population based multicenter study including 123 MEN1 patients with PA by de Laat et al. was at lowest risk of bias. This study showed that prolactinomas in MEN1 patients respond well to medical treatment. Furthermore, this study showed that tumor growth was very limited over time and almost always without clinical consequences. In contrast, Verges et al. found a significant difference in normalization of pituitary hypersecretion between MEN1 and non-MEN1 functional PA (42 vs. 90%, respectively, *P* < 0.001). Normalization of plasma prolactin was significantly less frequent in MEN1 (44%) vs. non-MEN1 patients (90%) (*P* < 0.001). Salenave et al. reported the presence of a *MEN1* mutation as a significant and independent predictor of dopamine agonist resistance in a regression analysis of 77 patients with prolactinoma (*t* = 3.052, *P* = 0.004). However, in this study a low number of MEN1 patients (*n* = 3) was included.

Treatment outcome in patients with *Xq26.3* microduplications (also known as X-Linked Acrogigantism, or X-LAG) is described in three studies (14, 17, 61). Since *Xq26.3* microduplications lead to an excessive growth velocity in the first years of life, X-LAG patients have a younger age at diagnosis and younger age at therapy-induced hormonal control than non-mutated counterparts (14). Due to this distinctive phenotype, it is hard to compare these results with other (sporadic) patients with PA. The proportion of patients in which disease control was reached varied due to the use of different definitions (41.7–91.7%). Multimodal treatment was necessary in the majority of cases, and hypopituitarism occurred frequently (70.6–75%). Hormonal control could almost never be achieved by medical therapy (dopamine agonists or SSA) alone (61). When comparing treatment outcome with pituitary induced gigantism without genetic abnormalities, Rostomyan et al. and Iacovazzo et al. found no differences in number of treatment modalities or prevalence of hypopituitarism between groups. The percentage of patients with long-term disease control (>12 months) did not differ significantly (X-LAG: 41.7%, controls: 38.4%), but appropriate control of GH/IGF-1 levels at last follow-up was reached more frequently in X-LAG patients (58.0 vs. 43.0%, *P* = 0.02) (14). For more study results, see Supplementary Data Sheet 5.

No eligible studies were found on the implications of germline mutations in *PRKAR1A, CDKN1B*, and *SDHx*.

## Discussion

The prevalence of germline mutations in unselected sporadically occurring PA is low. Therefore, germline analysis is not advisable for all patients. Based on the best-available evidence, the best predictor of an *AIP* or *MEN1* mutation appears to be a younger age at diagnosis (≤30 years). Moreover, the prevalence of an *AIP* mutation is significantly higher in pediatric patients in comparison to young adults (13, 26, 45).

Focusing on *AIP* mutations, the presence of gigantism and macroadenoma seems to be additional predictors of these mutations. The overgrowth may be attributed to the effect of GH/IGF-1 excess before full bone maturation. A male predominance in *AIP* affected individuals was found in a number studies (13, 26, 55). However, since it is conceivable that men are more prone to gigantism due to later growth cessation and male predominance was not observed in large families with an *AIP* mutation, this phenomenon might be explained by ascertainment bias (33). Both younger age at diagnosis and macroadenoma can be an expression of a more aggressive course of *AIP* mutation related PAs. Data on other factors such as adenoma subtype or the extent of tumor expansion are conflicting or too limited to draw clear conclusions.

*MEN1* mutation analysis is recommended in young patients (≤30 years). In one study, it is even suggested that *MEN1* mutations are more frequently found in prolactinomas (13). However, this is not yet confirmed in other studies.

Given the relatively high disease burden and younger age, patients suffering from pituitary related gigantism constitute a separate category. Germline *Xq26.3* microduplications were strongly associated with an early increased growth velocity and female gender. Since all reported patients harboring *Xq26.3* microduplication experienced a start of rapid growth already below 5 years of age, it is reasonable to perform genetic analysis for Xq26.3 microduplications especially in this subset of patients with sporadic pituitary gigantism (14, 16, 17, 61).

No cases of germline mutations in the *PRKAR1A* gene, *SDHx* genes, and *CDKN1B* or *CDKN2C* gene were reported in the included articles, which can be explained by our focus on apparently sporadically occurring PA instead of PA occurring with other syndromic manifestations. In addition, PA only very rarely occurs as manifestation of these, also rare, genetic syndromes. Therefore, genetic analysis of *PRKAR1A, SDHx*, and *CDKN1B* should only be conducted in selected cases with suggestive (syndromic) features.

*AIP* mutated somatotroph adenomas are more frequently resistant to SSA treatment than their non-mutated counterparts and reoperation is needed more often. Low *AIP* protein expression in tissue is correlated with worse response to SSA treatment (66), but since *AIP* downregulation may occur regardless of *AIP* mutations, it is still uncertain which mechanisms are involved (67). Failure of response to dopamine treatment is also described frequently in *AIP* mutation associated prolactinoma (9, 26). Treatment outcome seems similar when comparing study results of cohorts of sporadic and mainly familial occurring *AIP* mutation related PA patients, but data are too limited to draw clear conclusions (9, 26). Multimodal treatment is needed regularly but comparable with the treatment modalities in non-mutated controls, and difference in disease control did not reach statistical significance (9). There are too little reliable comparative data to determine the influence of an *AIP* mutation on treatment outcome in NFPA.

Best available evidence shows that *MEN1* mutation associated prolactinomas respond well to medical treatment and NFPA show no to very little tumor growth in virtually all cases (62). These findings are in contrast with earlier findings (60), partially due to the population based cohort studied by de Laat et al. and the inclusion of PA diagnosed by screening (*n* = 66).

The presence of *Xq26.3* microduplication is not related to a different treatment outcome compared to other cases of pituitary gigantism. Nonetheless, multiple treatment modalities are needed in most patients and complications such as hypopituitarism are frequent (14, 17, 61). Due to scarcity of reported quantitative information on treatment outcome of PA associated with mutations in *PRKAR1A, CDKN1B*, and *SDHx*, the impact of these germline mutations on therapy and outcome could not be predicted. The summary of recommendations and findings is presented in Table 5.

**TABLE 5:** Summary of recommendations and findings.

**Recommendations for genetic testing**	**Quality of evidence^a^**	**Strength of recommendation^b^**
Genetic analysis should not be done routinely in patients with sporadic pituitary adenoma	Low	Strong
*AIP* mutation analysis is recommended in young (≤ 30 years at diagnosis) sporadic pituitary adenoma, especially in the presence of gigantism and macroadenoma	Low	Weak
*MEN1* mutation analysis is recommended in young (≤ 30 years at diagnosis) sporadic pituitary adenoma patients (mainly prolactinoma)	Low	Weak
Genetic analysis for *Xq26.3* microduplications must be considered in sporadic pituitary gigantism with early start of rapid growth (<5 years), especially in female	Very low	Weak
Mutation analysis of *CDKN1B, PRKAR1A* and *SDHx* genes is not recommended in sporadic non-syndromic pituitary adenoma	Low	Strong
**Summary of findings on treatment outcome**		
*AIP* associated somatotroph adenoma are more frequently resistant to somatostatin analog treatment than non-mutated controls. Multimodal treatment is needed frequently but comparable with non-mutated controls, difference in disease control did not reach statistical significance.		
There is some evidence that treatment outcome is better in *AIP* associated gigantism, but given the considerable risk of bias and limited publications, no well-founded conclusions can be drawn for this subgroup.		
Failure of dopamine agonists is described frequently in *AIP* associated prolactinoma, and multimodal treatment is necessary in the majority of cases. There are too little reliable comparative data to determine the influence of an *AIP* mutation on treatment outcome in prolactinoma.		
*MEN1* associated prolactinoma respond well to dopamine agonist treatment and tumor growth of NFPA is often without clinical consequences.		
Treatment is challenging in X-LAG patients given the frequent use of multiple modalities and the occurrence of hypopituitarism. No significantly difference in long-term disease control, hypopituitarism, and the number of treatments is reported between X-LAG and other pituitary induced gigantism patients.		
Due to scarcity of reported quantitative data on treatment outcome of pituitary adenoma in Carney Complex, MEN4 and patients with *SDHx* mutations, it turned out to!!break be impossible to draw well-founded conclusions on the impact of these germline mutations		

*NFPA, non-functioning pituitary adenoma; X-LAG, X-Linked Acrogigantism*.

*Evidence Grading of Recommendations, Assessment, Development, and Evaluation (GRADE)*.

a *Quality of evidence (scale): High, Moderate, Low, Very Low*.

b *Strength of recommendation (scale): Strong, Weak*.

The majority of studies showed a considerable risk of bias, which can be partially explained by small study sizes inherent to the rarity of the disease. Most of the reported study populations were included in a non-random and non-consecutive manner and study cohorts were frequently selected from tertiary care centers, leading to potential patient selection bias. In some, mostly older studies, genetic analysis was not performed according to current quality standards. Furthermore, classification of genetic variants regarding the appropriate level of pathogenicity did not always take place according to the American College of Medical Genetics and Genomics and Association for Molecular Pathology (AMCG-AMP) guidelines (68). These genetic issues introduce a risk of detection bias. The retrospective design and lack of standardized data collection in most studies further hamper the methodological quality. Moreover, it cannot be excluded that parts of included study cohorts were reported previously, introducing a possible distortion in results. Therefore, results must be interpreted with caution before drawing conclusions and especially before being used for decision making in daily clinical practice.

Still, the aim of this review was to retrieve highly applicable best-available evidence on specific clinically relevant questions. Although we attempted to retrieve additional results, insufficient reporting of outcomes concerning our predefined topics led to exclusion of otherwise valuable records. We did exclude too small sized studies to avoid imprecise estimations. In addition, we did not perform a meta-analysis of data because of the high heterogeneity of studies to avoid unreliable outcomes. Additionally, we used the presented results on the adenoma subtype as described in the individual papers, because immunochemistry results were not always provided. This could have resulted in slightly inaccurate results in NFPA, since immunostaining can reveal clinically silent or “whispering” adenomas with some evidence of biochemical hypersecretion. Given the distinctive clinical behavior of these subtypes, a thorough investigation of adenoma subtype according to the most recent World Health Organization guidelines would have provided us with more accurate results (69, 70). However, we provided all available data on immunohistochemistry of NFPA in the results tables. Finally, the large range of publication dates introduced a challenge in the interpretation of pathogenicity of genetic variants. By adopting the author's judgement, outdated knowledge or techniques can have resulted in inaccuracy of the results. Optimally, all historic results would have to be confirmed by the current standards of DNA analysis and interpretation. Therefore, the DNA analysis techniques and interpretation of genetic variants (e.g., loss of heterozigosity studies, worldwide SNP databases, *in silico* analysis, functional studies) were evaluated thoroughly in our critical appraisal to put the results into the right perspective.

In general, our results support earlier findings and reviews on genetic analysis in PA (71–74). Recently, Caimari et al. developed a user-friendly risk category system to find *AIP* mutation associated PA using a large international cohort of 2,227 individuals. Young age of onset, familial status, GH excess, and macroadenoma were the strongest predictors (65). However, in contrast to these study results and earlier reviews, our recommendations are focused on apparently sporadically occurring PA in patients without other features of genetic syndromes. Furthermore, they come with the proper strength of recommendations as a result of the systematic literature search and critical appraisal of articles.

A number of unanswered questions and challenges for the future still remain. As a result of the rarity of diseases and/or PA as presenting manifestation, the clinical impact of a *CDKN1B, PRKAR1A*, and *SDHx* mutations on treatment outcome of PA is still uncertain. Only worldwide networks of collaborating centers sharing clinical information can help unravel this issue. Secondly, the implications of an *AIP* mutation in apparent unaffected family members are unknown. To our knowledge, results from systematic follow-up of unaffected *AIP*-positive family members are not available. Therefore, surveillance guidelines in these cases await further studies. Furthermore, the number of germline variants of uncertain significance will continue to increase in the (near) future due to the increased genetic analysis modalities, further emphasizing the need for studies of functional status combined with data on clinical outcome from large worldwide databases. Lastly, despite our efforts to produce reliable recommendations, it remains difficult to predict the benefits of our recommendations when implementing them in daily practice. For example, in a recent study by Daly et al., no germline mutations in the *AIP* or *MEN1* gene were identified in a group of 55 PA patients, despite the use of risk criteria (75). These results show that no risk stratification system or set of screening recommendations is flawless. By external validation and further (clinical) research these tools can be optimized in the future, but will never be all comprehensive.

Based on the yet available literature on the value of genetic analysis of sporadic PA, we can conclude that effective use of genetic analysis can lead to early disease identification (with possibly beneficial treatment outcome) on the one hand, and can lower health care costs and psychological burden on the other hand if unnecessary investigations can be limited. Knowledge of the effect of germline mutations on treatment outcome helps to determine therapy strategy and possibly lowers disease morbidity. Now, large and unselected cohort studies, are needed to further guide the indications and the consequences of mutation analysis in individual patients with PA.

## Author Contributions

MB, RL, and GV contributed to conception and design of the study. MB and BN contributed to data collection (selection of articles) and contributed to the critical appraisal of studies. MB contributed to data extraction and wrote the first draft of the manuscript. MB, BN, AV, RL, and GV contributed to the interpretation of data. RL and GV contributed to supervision of data collection, supervision of critical appraisal of studies, and supervison of data extraction. All authors contributed to critically reviewing the manuscript, read, and approved the submitted version.

### Conflict of Interest

The authors declare that the research was conducted in the absence of any commercial or financial relationships that could be construed as a potential conflict of interest.
