# Supplementary material for: Clinical Relevance of Genetic Analysis in Patients With Pituitary Adenomas: A Systematic Review
Source: Front Endocrinol (Lausanne). 2019 Dec 10;10:837. doi: 10.3389/fendo.2019.00837 (PMC6914701; doi:10.3389/fendo.2019.00837)
Supplement: Supplementary file 1 [file Data_Sheet_1.docx]

**Supplemental Material 1: Search strategy**

**1. Graphic search overview**

AND

OR

Prolactinoma

Prolactinomas

Lactotroph adenoma

Lactotroph adenomas

Somatotropinoma

Somatotropinomas

Somatotroph adenoma

Somatotroph adenomas

GH-producing adenoma

GH producing adenoma

GH-producing adenomas

GH producing adenomas

Corticotroph adenoma

Corticotroph adenomas

ACTH-producing adenoma

ACTH producing adenoma

ACTH-producing adenomas

ACTH producing adenomas

Thyrotroph adenoma

Thyrotroph adenomas

non-functioning pituitary adenoma

non functioning pituitary adenoma

non-functioning pituitary adenomas

non functioning pituitary adenomas

FSH-secreting adenoma

FSH secreting adenoma

FSH-secreting adenomas

FSH secreting adenomas

TSH producing adenoma

TSH-producing adenoma

TSH-producing adenomas

TSH producing adenomas

NFPA

NFPAs

NFA

NFAs

AND

adenoma

adenomas

tumor

tumors

tumour

tumours

macroadenoma

macroadenomas

microadenoma

microadenomas

neoplasm

neoplasms

incidentaloma

incidentalomas

mass

masses

Pituitary

PRKAR1A

PRKACA

PRKACB

Carney complex

GPR101

XLAG

X LAG

X-LAG

X linked acrogigantism

X-linked acrogigantism

SDHx

SDHA

SDHB

SDHC

SDHD

3PAs

Genetic analysis

Genetic counseling

Genetic testing

Genetic screening

Genetics

Mutation

Mutations

Gene expression

AIP

aryl-hydrocarbon receptor-interacting protein

aryl hydrocarbon receptor-interacting protein

aryl-hydrocarbon receptor interacting protein

aryl hydrocarbon receptor interacting protein

MEN1

MEN4

CDKN1B

Multiple endocrine neoplasia

**2. Pubmed search**

#1 pituitary[Title/Abstract]

# 2 (((((((((((((((adenoma[Title/Abstract]) OR adenomas[Title/Abstract]) OR tumor[Title/Abstract]) OR tumors[Title/Abstract]) OR tumour[Title/Abstract]) OR tumours[Title/Abstract]) OR macroadenoma[Title/Abstract]) OR macroadenomas[Title/Abstract]) OR microadenoma[Title/Abstract]) OR microadenomas[Title/Abstract]) OR neoplasm[Title/Abstract]) OR neoplasms[Title/Abstract]) OR incidentaloma[Title/Abstract]) OR incidentalomas[Title/Abstract]) OR mass[Title/Abstract]) OR masses[Title/Abstract]

#3 #1 AND #2

#4 (((((((((((((((((((((((((((((((((((prolactinoma[Title/Abstract]) OR prolactinomas[Title/Abstract]) OR "lactotroph adenoma"[Title/Abstract]) OR "lactotroph adenomas"[Title/Abstract]) OR somatotropinoma[Title/Abstract]) OR somatotropinomas[Title/Abstract]) OR "somatotroph adenoma"[Title/Abstract]) OR "somatotroph adenomas"[Title/Abstract]) OR "GH-producing adenoma"[Title/Abstract]) OR "GH-producing adenomas"[Title/Abstract]) OR "GH producing adenoma"[Title/Abstract]) OR "GH producing adenomas"[Title/Abstract]) OR "corticotroph adenoma"[Title/Abstract]) OR "corticotroph adenomas"[Title/Abstract]) OR "ACTH-producing adenoma"[Title/Abstract]) OR "ACTH-producing adenomas"[Title/Abstract]) OR "ACTH producing adenoma"[Title/Abstract]) OR "ACTH producing adenomas"[Title/Abstract]) OR "thyrotroph adenoma"[Title/Abstract]) OR "thyrotroph adenomas"[Title/Abstract]) OR "TSH producing adenoma"[Title/Abstract]) OR "TSH producing adenomas"[Title/Abstract]) OR "TSH-producing adenoma"[Title/Abstract]) OR "TSH-producing adenomas"[Title/Abstract]) OR "FSH-secreting adenoma"[Title/Abstract]) OR "FSH-secreting adenomas"[Title/Abstract]) OR "FSH secreting adenoma"[Title/Abstract]) OR "FSH secreting adenomas"[Title/Abstract]) OR NFPA[Title/Abstract]) OR NFPAs[Title/Abstract]) OR NFA[Title/Abstract]) OR NFAs[Title/Abstract]) OR "non-functioning pituitary adenoma"[Title/Abstract]) OR "non-functioning pituitary adenomas"[Title/Abstract]) OR "non functioning pituitary adenoma"[Title/Abstract]) OR "non functioning pituitary adenomas"[Title/Abstract]

#5 #3 OR #4

#6 (((((((((((((((((((((((((((((((("genetic analysis"[Title/Abstract]) OR "genetic counseling"[Title/Abstract]) OR "genetic testing"[Title/Abstract]) OR "genetic screening"[Title/Abstract]) OR genetics[Title/Abstract]) OR mutation[Title/Abstract]) OR mutations[Title/Abstract]) OR "gene expression"[Title/Abstract]) OR AIP[Title/Abstract]) OR "aryl-hydrocarbon receptor-interacting protein"[Title/Abstract]) OR "aryl-hydrocarbon receptor interacting protein"[Title/Abstract]) OR "aryl hydrocarbon receptor-interacting protein"[Title/Abstract]) OR "aryl hydrocarbon receptor interacting protein"[Title/Abstract]) OR MEN1[Title/Abstract]) OR MEN4[Title/Abstract]) OR "multiple endocrine neoplasia"[Title/Abstract]) OR CDKN1B[Title/Abstract]) OR PRKAR1A[Title/Abstract]) OR PRKACA[Title/Abstract]) OR PRKACB[Title/Abstract]) OR "Carney complex"[Title/Abstract]) OR GPR101[Title/Abstract]) OR XLAG[Title/Abstract]) OR "X LAG"[Title/Abstract]) OR "X-LAG"[Title/Abstract]) OR "X linked acrogigantism"[Title/Abstract]) OR "X-linked acrogigantism"[Title/Abstract]) OR SDHx[Title/Abstract]) OR SDHA[Title/Abstract]) OR SDHB[Title/Abstract]) OR SDHC[Title/Abstract]) OR SDHD[Title/Abstract]) OR 3PAs[Title/Abstract]

#7 #5 AND #6

Limits: Humans, Language (English; Dutch; French; German)

**Search result 22^th^ of November 2018: 2017**

**3. Embase search**

#1 pituitary:ab,ti

#2 'adenoma':ab,ti OR 'adenomas':ab,ti OR 'tumor':ab,ti OR 'tumors':ab,ti OR 'tumour':ab,ti OR 'tumours':ab,ti OR 'macroadenoma':ab,ti OR 'macroadenomas':ab,ti OR 'microadenoma':ab,ti OR 'microadenomas':ab,ti OR 'neoplasm':ab,ti OR 'neoplasms':ab,ti OR 'incidentaloma':ab,ti OR 'incidentalomas':ab,ti OR 'mass':ab,ti OR 'masses':ab,ti

#3 #1 AND #2

#4 'prolactinoma':ab,ti OR 'prolactinomas':ab,ti OR 'lactotroph adenoma':ab,ti OR 'lactotroph adenomas':ab,ti OR 'somatotropinoma':ab,ti OR 'somatotropinomas':ab,ti OR 'somatotroph adenoma':ab,ti OR 'somatotroph adenomas':ab,ti OR 'GH-producing adenoma':ab,ti OR 'GH-producing adenomas':ab,ti OR 'GH producing adenoma':ab,ti OR 'GH producing adenomas':ab,ti OR 'corticotroph adenoma':ab,ti OR 'corticotroph adenomas':ab,ti OR 'ACTH-producing adenoma':ab,ti OR 'ACTH-producing adenomas':ab,ti OR 'ACTH producing adenoma':ab,ti OR 'ACTH producing adenomas':ab,ti OR 'thyrotroph adenoma':ab,ti OR 'thyrotroph adenomas':ab,ti OR 'TSH producing adenoma':ab,ti OR 'TSH producing adenomas':ab,ti OR 'TSH-producing adenoma':ab,ti OR 'TSH-producing adenomas':ab,ti OR 'FSH-secreting adenoma':ab,ti OR 'FSH-secreting adenomas':ab,ti OR 'FSH secreting adenoma':ab,ti OR 'FSH secreting adenomas':ab,ti OR 'NFPA':ab,ti OR 'NFPAs':ab,ti OR 'NFA':ab,ti OR 'NFAs':ab,ti OR 'non-functioning pituitary adenoma':ab,ti OR 'non-functioning pituitary adenomas':ab,ti OR 'non functioning pituitary adenoma':ab,ti OR 'non functioning pituitary adenomas':ab,ti

#5 #3 OR #4

#6 'genetic analysis':ab,ti OR 'genetic counseling':ab,ti OR 'genetic testing':ab,ti OR 'genetic screening':ab,ti OR 'genetics':ab,ti OR 'mutation':ab,ti OR 'mutations':ab,ti OR 'gene expression':ab,ti OR 'AIP':ab,ti OR 'aryl-hydrocarbon receptor-interacting protein':ab,ti OR 'aryl-hydrocarbon receptor interacting protein':ab,ti OR 'aryl hydrocarbon receptor-interacting protein':ab,ti OR 'aryl hydrocarbon receptor interacting protein':ab,ti OR 'MEN1':ab,ti OR 'MEN4':ab,ti OR 'multiple endocrine neoplasia':ab,ti OR 'CDKN1B':ab,ti OR 'PRKAR1A':ab,ti OR 'PRKACA':ab,ti OR 'PRKACB':ab,ti OR 'Carney complex':ab,ti OR 'GPR101':ab,ti OR 'XLAG':ab,ti OR 'X LAG':ab,ti OR 'X-LAG':ab,ti OR 'X linked acrogigantism':ab,ti OR 'X-linked acrogigantism':ab,ti OR 'SDHx':ab,ti OR 'SDHA':ab,ti OR 'SDHB':ab,ti OR 'SDHC':ab,ti OR 'SDHD':ab,ti OR '3PAs':ab,ti

#7 #5 AND #6

Limits: Embase, Humans, Language (English; Dutch; French; German)

**Search result 22^th^ of November 2018: 2673**

**4. Web of Science search**

#1 TS=pituitary

#2 TS=(adenoma OR adenomas OR tumor OR tumors OR tumour OR tumours OR macroadenoma OR macroadenomas OR microadenoma OR microadenomas OR neoplasm OR neoplasms OR incidentaloma OR incidentalomas OR mass OR masses)

#3 #1 AND #2

#4 TS=(prolactinoma OR prolactinomas OR “lactotroph adenoma” OR “lactotroph adenomas” OR somatotropinoma OR somatotropinomas OR “somatotroph adenoma” OR “somatotroph adenomas” OR “GH-producing adenoma” OR “GH-producing adenomas” OR “GH producing adenoma” OR “GH producing adenomas” OR “corticotroph adenoma” OR “corticotroph adenomas” OR “ACTH-producing adenoma” OR “ACTH-producing adenomas” OR “ACTH producing adenoma” OR “ACTH producing adenomas” OR “thyrotroph adenoma” OR “thyrotroph adenomas” OR “TSH producing adenoma” OR “TSH producing adenomas” OR “TSH-producing adenoma” OR “TSH-producing adenomas” OR “FSH-secreting adenoma” OR “FSH-secreting adenomas” OR “FSH secreting adenoma” OR “FSH secreting adenomas” OR NFPA OR NFPAs OR NFA OR NFAs OR “non-functioning pituitary adenoma” OR “non-functioning pituitary adenomas” OR “non functioning pituitary adenoma” OR “non functioning pituitary adenomas”)

#5 #3 OR #4

#6 TS=(“genetic analysis” OR “genetic counseling” OR “genetic testing” OR “genetic screening” OR genetics OR mutation OR mutations OR “gene expression” OR AIP OR “aryl-hydrocarbon receptor-interacting protein” OR “aryl-hydrocarbon receptor interacting protein” OR “aryl hydrocarbon receptor-interacting protein” OR “aryl hydrocarbon receptor interacting protein” OR MEN1 OR MEN4 OR “multiple endocrine neoplasia” OR CDKN1B OR PRKAR1A OR PRKACA OR PRKACB OR “Carney complex” OR GPR101 OR XLAG OR “X LAG” OR “X-LAG” OR “X linked acrogigantism” OR “X-linked acrogigantism” OR SDHx OR SDHA OR SDHB OR SDHC OR SDHD OR 3PAs)

#7 #5 AND #6

Limits: Language (English; French; German)

**Search result 22^th^ of November 2018: 4412**
