# Supplementary material for: Clinical Relevance of Genetic Analysis in Patients With Pituitary Adenomas: A Systematic Review
Source: Front Endocrinol (Lausanne). 2019 Dec 10;10:837. doi: 10.3389/fendo.2019.00837 (PMC6914701; doi:10.3389/fendo.2019.00837)
Supplement: Supplementary file 2 [file Data_Sheet_2.docx]

**Supplemental Material 2 Excluded studies**

***Predictors on germline mutation status in sporadic PA***

| **Author** | **Year** | **Main reason of exclusion** | **Explanation** |
| --- | --- | --- | --- |
| Corbetta (1) | 1997 | Syndromic phenotype | Genetic analysis was only performed in patients with additional MEN1 related features. |
| Raitila (2) | 2007 | Not clear if sporadic | The family history of one of the two patients with an *AIP* germline mutation has been reported as ‘not available’. |
| Georgitsi (3) | 2008 | Only MLPA used | This study only reported data on multiplex ligation-dependent probe amplification (MLPA) studies. |
| Igreja (4) | 2009 | Syndromic phenotype | The study cohort consisted of patients with a clinical MEN1 syndrome, who tested negative for mutations in the *MEN1* gene. |
| Daly (5) | 2010 | Not clear if sporadic | This study focused on the clinical and therapeutic features of patients with an *AIP* germline mutation. The study population consisted of both sporadic and familial cases. It was not possible to investigate the sporadic cases separately. |
| Cazabat (6) | 2011 | Cohort duplicate | All patients reported in this article were included in a study by Cazabat *et al.* in 2012 and Lecoq *et al*. in 2016. (7,8) |
| Nozières (9) | 2011 | < 5 patients included | Only four patients with sporadic pituitary adenoma were described in this study. |
| Cazabat (7) | 2012 | Cohort duplicate | All study participants were included in the study by Lecoq *et al.* in 2016. (8) |
| Belar (10) | 2012 | Not clear if sporadic | 79 patients, both sporadic and familial, were included in this study. It was not possible to investigate the sporadic cases separately. |
| Papathomas (11) | 2014 | Not clear if sporadic | The familial status of the six included patients has been reported as unknown. |
| Demir (12) | 2014 | Pathogenicity of investigated genes unclear | This study focused on germline loss-of-function mutations in *inhibitory guanine nucleotide (GTP) binding protein alpha (GNAI)* loci. There is insufficient evidence in literature for *GNAI* allelic variants in the tumorigenesis of PA. |
| Nunes (13) | 2014 | Not clear if sporadic | The family history of included patients was only retrieved when *MEN1* mutation analysis showed a positive result. |
| Beckers (14) | 2015 | Cohort duplicate | All 13 sporadic patients suffering from X-LAG have been included in the study by Trivellin *et al.* in 2014. (15) |
| Salenave (16) | 2015 | Not clear if sporadic | The familial status of the study participants has not been described. |
| Ye (17) | 2015 | Pathogenicity of investigated genes unclear | This paper described a genome wide association study. |
| Denes (18) | 2015 | Syndromic phenotype | The study population consisted of patients with (1) both pheochromocytoma/paraganglioma and pituitary adenoma, and (2) the occurrence of pheochromocytoma/paraganglioma and pituitary adenoma within their family. |
| Rostomyan (19) | 2015 | Not clear if sporadic | The study population consisted of both sporadic and familial cases. It was not possible to investigate the sporadic cases separately. |
| Xekouki (20) | 2015 | Syndromic phenotype | This study focused on the co-occurrence of pituitary adenoma and pheochromocytoma/paraganglioma. |
| Hu (21) | 2016 | Pathogenicity of investigated genes unclear | This study focused on the possible role of single nucleotide polymorphisms (SNP) within the *AIP* gene in non‑functioning pituitary adenomas. |
| Iacovazzo (22) | 2016 | Not clear if sporadic | The study cohort of acromegaly patients included both sporadic and familial cases. It was not possible to investigate the sporadic cases separately. |
| Peculis (23) | 2016 | Pathogenicity of investigated genes unclear | This study focused on the possible role of single nucleotide polymorphisms (SNP) in seven genes in pituitary adenomas. |
| Ramírez-Rentería (24) | 2016 | Cohort duplicate | 70 out of 71 patients have been described previously in a study by Leontiou *et al.* (25) |
| Trivellin (26) | 2016 | Pathogenicity of investigated genes unclear | This research focused on allelic variants of the *GPR101* gene. |
| Zhang (27) | 2017 | Pathogenicity of investigated genes unclear | This research focuses on genetic variations in the *cadherin-*  *related 23 (CDH23*) gene in pituitary adenoma patients. There is insufficient evidence in literature to date for its role in the tumorigenesis of PA. |
| Hernández-Ramírez (28) | 2017 | Pathogenicity of investigated genes unclear | The authors investigated the presence of *CABLES1* mutations/copy number variations (CNVs) in pediatric cases of ACTH producing pituitary adenoma. There is insufficient evidence in literature to date for its role in the tumorigenesis of PA. |
| Hernández-Ramírez (29) | 2017 | Not clear if sporadic | This study reported a case of Cushing disease due to a loss-of-function  mutation in PRKAR1A and reported a lack of mutations in 97 other pediatric Cushing patients. The familial status of these patients is unknown. |
| Radian (30) | 2017 | Cohort duplicate | This study focused on carrier frequency of *AIP* founder mutation R304∗ in specific North Ireland and Ireland regions. The R304∗-positive patients were previously reported in several studies (i.a. Leontiou *et al*. 2008, Hernandez-Ramirez *et al*. 2015) (25,31) |
| Iivonen (32) | 2018 | Pathogenicity of investigated genes unclear | This work focused on germline mutations in the potassium voltage-gated channel subfamily Q member 1 (*KCNQ1*) and potassium voltage-gated channel subfamily E regulatory subunit 2 (KCNE2) gene. There is insufficient evidence in literature to date for its role in the tumorigenesis of PA. |
| Caimari (33) | 2018 | Not clear if sporadic | The authors presented a new risk category system for *AIP* mutations in patients with pituitary adenomas. The study population consisted of both sporadic and familial cases. It was not possible to investigate the sporadic cases separately. |
| Nagata (34) | 2018 | Not clear if sporadic | The study cohort included both sporadic and familial cases. It was not possible to investigate the sporadic cases separately. |
| Makri (35) | 2018 | Not clear if sporadic | This retrospective analysis of paediatric patients with Cushing disease included both sporadic and familial cases. It was not possible to investigate the sporadic cases separately. |

***Impact of a germline mutation on treatment outcome in pituitary adenoma***

| **Author** | **Year** | **Main reason of exclusion** | **Explanation** |
| --- | --- | --- | --- |
| Scheithauer (36) | 1987 | No germline mutation analysis | Surgically resected pituitary adenomas in the setting of multiple endocrine neoplasia type 1 were compared with pituitary adenomas occurring in the general population. There was no germline mutation analysis performed in this study. |
| Burgess (37) | 1996 | No germline mutation analysis | Prolactinomas in a large kindred with multiple endocrine neoplasia type 1 were compared with literature. There was no germline mutation analysis performed in this study. |
| Burgess (38) | 1996 | No germline mutation analysis | Clinical, biochemical and radiological features of pituitary disease in a large MEN1 kindred were described. There was no germline mutation analysis performed in this study. |
| Vierimaa (39) | 2006 | No information on treatment outcome | Vierimaa *et al*. describe the discovery of the *AIP* gene. Age at diagnosis, gender,  and size of adenoma were compared between seven patients with a germline *AIP* mutation and patients without *AIP* mutation. No information on treatment outcome was reported. |
| Daly (40) | 2007 | No information on treatment outcome | 73 FIPA families are described in this study. Age at diagnosis, gender,  and size of adenoma were compared between seven patients with a germline *AIP* mutation and patients without *AIP* mutation. No information on treatment outcome was reported. |
| Cazabat (41) | 2007 | No information on treatment outcome | Age at diagnosis, gender and patient length is compared in *AIP* mutated and non-*AIP* mutated patients with sporadic GH secreting pituitary adenoma. Insufficient information on treatment outcome was reported. |
| Trouillas (42) | 2008 | No information on treatment outcome | Pituitary tissue specimens of 77 MEN1 patients were compared with unselected  2509 non-MEN1 sporadic pituitary tumors. There was no information about treatment outcome reported. |
| Leontiou (25) | 2008 | No individual data is available | 26 FIPA families and 85 patients with sporadic pituitary adenoma were described. Treatment outcome of families with a germline *AIP* mutation was described, but no individual data was presented. |
| Igreja (43) | 2010 | No information on treatment outcome | 64 FIPA families are described. Age and penetrance are compared between *AIP* mutated and non-mutated patients. There was no information about treatment outcome reported. |
| Cain (44) | 2010 | Cohort duplicate | The study cohort was identical to the study population reported in Igreja *et al.* in 2010*.* (43) |
| De Pinho (45) | 2010 | Cohort duplicate | Four FIPA families were described, of which one with an *AIP* germline mutation.Three out of four families were reported previously in Igreja *et al*. in 2010. (43) |
| Cazabat (7) | 2012 | No information on treatment outcome | 443 cases with apparent sporadic pituitary adenoma, of which 16 with *AIP* germline mutation, are described. No information on treatment outcome is reported. |
| Vroonen (46) | 2012 | No clinical data on subgroup of patients harboring germline mutation | This study focused on 92 cabergoline resistent prolactinoma. Patients with a ‘genetic basis’ were compared with the rest of the study population. This group included patients with a germline *MEN1* or *AIP* mutation, an *AIP* variant of unknown significance (VUS), familial disease (FIPA) without germline mutation/variant, and one clinical MEN1 patient without germline mutation. No clinical data on the subgroup of patients harboring germline mutation were provided. |
| Simonds (47) | 2012 | < 5 cases with germline mutation | Only four patients with a germline mutation (*MEN1*) were included in this study. |
| Oriola (48) | 2013 | < 5 cases with germline mutation | Only two patients with *AIP* germline mutation (and two patients with a *AIP* gene VUS) were included in this study. |
| Cuny (49) | 2013 | No information on treatment outcome | 174 patients with sporadic pituitary macroadenoma were described. There was no information about treatment outcome reported. |
| Trivellin (15) | 2014 | Cohort duplicate | Clinical and biochemical characteristics of 43 patients with gigantism and GH producing pituitary adenoma were described. The discovery of *Xq26.3* genomic duplication and the related phenotype of X-LAG (14 patients) was described.  Thirteen out of 14 XLAG patients have been reported in Beckers *et al*. in 2015. (14) |
| Preda (50) | 2014 | < 5 cases with germline mutation | Only four patients with a germline mutation were included in this study. |
| Schöfl (51) | 2014 | No clinical information on subgroup of patients harboring germline mutation | 91 young patients with acromegaly were described, of which five harboring an *AIP* germline mutation. No clinical information about the subgroup of this patients are presented. |
| Hernandez-Ramirez (31) | 2015 | No information on treatment outcome | In this study the *AIP* mutation status and clinical characteristics of 216 FIPA families and 404 sporadic pituitary adenoma patients were described. However, there was no information about the relationship between germline mutation status and treatment outcome reported. |
| Ramírez-Rentería (24) | 2016 | Cohort duplicate | 70 out of 71 patients were described previously in Hernandez-Ramirez *et al*. in 2015. |
| Mangupli (52) | 2016 | < 5 cases with germline mutation | Only three patients with a germline mutation were included in this study. |
| Matsumoto (53) | 2016 | < 5 cases with germline mutation | Only three patients with a germline mutation were included in this study. |
| Lecoq (8) | 2016 | No information on treatment outcome | 766 patients with sporadic pituitary adenoma were described. Gender, age of diagnosis, gigantism y/n were compared between subgroups (*AIP* mutations or rare variants, *GPR101* variants, wildtype). No information about treatment outcome has been described. |
| Radian (30) | 2017 | Cohort duplicate | This study focused on carrier frequency of *AIP* founder mutation R304∗ in specific North Ireland and Ireland regions. The R304∗-positive patients have been reported previously in several studies (i.a. Leontiou *et al*. 2008, Hernandez-Ramirez *et al*. 2015) (25,31) |
| De Sousa (54) | 2017 | < 5 cases with germline mutation | Only four patients with a (likely) pathogenic germline variant were included in this study. |
| Ozkaya (55) | 2018 | < 5 cases with germline mutation | Only two patients with a germline mutation were included in this study. |
| Marques (56) | 2018 | Congress abstract | In this congress abstract presented at the 100th Annual Meeting of the Endocrine Society ENDO 2018, 184 pituitary adenoma patients with a germline *AIP* mutation were compared with non-mutated pituitary adenoma patients. However, the data has not been published in a full article yet. |

**References**

1. Corbetta S, Pizzocaro A, Peracchi M, Beck-Peccoz P, Faglia G, Spada A. Multiple endocrine neoplasia type 1 in patients with recognized pituitary tumours of different types. Clin Endocrinol (Oxf). 1997 Nov;47(5):507–12.

2. Raitila A, Georgitsi M, Karhu A, Tuppurainen K, Mäkinen MJ, Birkenkamp-Demtröder K, et al. No evidence of somatic aryl hydrocarbon receptor interacting protein mutations in sporadic endocrine neoplasia. Endocr Relat Cancer. 2007 Sep;14(3):901–6.

3. Georgitsi M, Heliovaara E, Paschke R, Kumar AVK, Tischkowitz M, Vierimaa O, et al. Large genomic deletions in AIP in pituitary adenoma predisposition. J Clin Endocrinol Metab. 2008 Oct;93(10):4146–51.

4. Igreja S, Chahal HS, Akker SA, Gueorguiev M, Popovic V, Damjanovic S, et al. Assessment of p27 (cyclin-dependent kinase inhibitor 1B) and aryl hydrocarbon receptor-interacting protein (AIP) genes in multiple endocrine neoplasia (MEN1) syndrome patients without any detectable MEN1 gene mutations. Clin Endocrinol (Oxf). 2009 Feb;70(2):259–64.

5. Daly AF, Tichomirowa MA, Petrossians P, Heliovaara E, Jaffrain-Rea M-L, Barlier A, et al. Clinical characteristics and therapeutic responses in patients with germ-line AIP mutations and pituitary adenomas: An international collaborative study. J Clin Endocrinol Metab. 2010 Nov;95(11):E373–83.

6. Cazabat L, Bouligand J, Chanson P. AIP Mutation in Pituitary Adenomas. N Engl J Med. 2011;364(20):1973–4.

7. Cazabat L, Bouligand J, Salenave S, Bernier M, Gaillard S, Parker F, et al. Germline AIP mutations in apparently sporadic pituitary adenomas: Prevalence in a prospective single-center cohort of 443 patients. J Clin Endocrinol Metab. 2012 Apr;97(4):E663–70.

8. Lecoq A-L, Bouligand J, Hage M, Cazabat L, Salenave S, Linglart A, et al. Very low frequency of germline GPR101 genetic variation and no biallelic defects with AIP in a large cohort of patients with sporadic pituitary adenomas. Eur J Endocrinol. 2016 Apr;174(4):523–30.

9. Nozières C, Berlier P, Dupuis C, Raynaud-Ravni C, Morel Y, Chazot FB, et al. Sporadic and genetic forms of paediatric somatotropinoma: a retrospective analysis of seven cases and a review of the literature. Orphanet J Rare Dis. 2011;6(1):67.

10. Belar O, De La Hoz C, Pérez-Nanclares G, Castano L, Gaztambide S. Novel mutations in MEN1, CDKN1B and AIP genes in patients with multiple endocrine neoplasia type 1 syndrome in Spain. Clin Endocrinol (Oxf). 2012 May;76(5):719–24.

11. Papathomas TG, Gaal J, Corssmit EPM, Oudijk L, Korpershoek E, Heimdal K, et al. Non-pheochromocytoma (PCC)/paraganglioma (PGL) tumors in patients with succinate dehydrogenase-related PCC-PGL syndromes: A clinicopathological and molecular analysis. Eur J Endocrinol. 2014 Jan;170(1):1–12.

12. Demir H, Donner I, Kivipelto L, Kuismin O, Schalin-Jäntti C, De Menis E, et al. Mutation analysis of inhibitory guanine nucleotide binding protein alpha (GNAI) loci in young and familial pituitary adenomas. PLoS One. 2014;9(10):e109897.

13. Nunes VS, Souza GL, Perone D, Conde SJ, Nogueira CR. Frequency of multiple endocrine neoplasia type 1 in a group of patients with pituitary adenoma: Genetic study and familial screening. Pituitary. 2014 Feb;17(6):30–7.

14. Beckers A, Lodish MB, Trivellin G, Rostomyan L, Lee M, Faucz FR, et al. X-linked acrogigantism syndrome: Clinical profile and therapeutic responses. Endocr Relat Cancer. 2015 Jun;22(3):353–67.

15. Trivellin G, Daly AF, Faucz FR, Yuan B, Rostomyan L, Larco DO, et al. Gigantism and Acromegaly Due to Xq26 Microduplications and *GPR101* Mutation. N Engl J Med. 2014;371(25):2363–74.

16. Salenave S, Ancelle D, Bahougne T, Raverot G, Kamenicky P, Bouligand J, et al. Macroprolactinomas in children and adolescents: Factors associated with the response to treatment in 77 patients. J Clin Endocrinol Metab. 2015 Mar;100(3):1177–86.

17. Ye Z, Li Z, Wang Y, Mao Y, Shen M, Zhang Q, et al. Common variants at 10p12.31, 10q21.1 and 13q12.13 are associated with sporadic pituitary adenoma. Nat Genet. 2015 Jul;47(7):793–8.

18. Denes J, Swords F, Rattenberry E, Stals K, Owens M, Cranston T, et al. Heterogeneous genetic background of the association of pheochromocytoma/paraganglioma and pituitary adenoma: Results from a large patient cohort. J Clin Endocrinol Metab. 2015 Mar;100(3):E531–41.

19. Rostomyan L, Daly AF, Petrossians P, Nachev E, Lila AR, Lecoq A-L, et al. Clinical and genetic characterization of pituitary gigantism: An international collaborative study in 208 patients. Endocr Relat Cancer. 2015 Oct;22(5):745–57.

20. Xekouki P, Szarek E, Bullova P, Giubellino A, Quezado M, Mastroyannis SA, et al. Pituitary adenoma with paraganglioma/pheochromocytoma (3PAs) and succinate dehydrogenase defects in humans and mice. J Clin Endocrinol Metab. 2015 May;100(5):E710–9.

21. Hu Y, Yang J, Chang Y, Ma S, Qi J. SNPs in the aryl hydrocarbon receptor-interacting protein gene associated with sporadic non-functioning pituitary adenoma. Exp Ther Med. 2016;11(3):1142–6.

22. Iacovazzo D, Caswell R, Bunce B, Jose S, Yuan B, Hernández-Ramírez LC, et al. Germline or somatic GPR101 duplication leads to X-linked acrogigantism: a clinico-pathological and genetic study. Acta Neuropathol Commun. 2016 Jun;4(1):56.

23. Peculis R, Balcere I, Rovite V, Megnis K, Valtere A, Stukens J, et al. Polymorphisms in MEN1 and DRD2 genes are associated with the occurrence and characteristics of pituitary adenomas. Eur J Endocrinol. 2016 Aug;175(2):145–53.

24. Ramírez-Rentería C, Hernández-Ramírez LC, Portocarrero-Ortiz L, Vargas G, Melgar V, Espinosa E, et al. AIP mutations in young patients with acromegaly and the Tampico Giant: the Mexican experience. Endocrine. 2016;53(2):402–11.

25. Leontiou CA, Gueorguiev M, van der Spuy J, Quinton R, Lolli F, Hassan S, et al. The role of the aryl hydrocarbon receptor-interacting protein gene in familial and sporadic pituitary adenomas. J Clin Endocrinol Metab. 2008 Jun;93(6):2390–401.

26. Trivellin G, Correa RR, Batsis M, Faucz FR, Chittiboina P, Bjelobaba I, et al. Screening for GPR101 defects in pediatric pituitary corticotropinomas. Endocr Relat Cancer. 2016;23(5):357–65.

27. Zhang Q, Peng C, Song J, Zhang Y, Chen J, Song Z, et al. Germline Mutations in CDH23, Encoding Cadherin-Related 23, Are Associated with Both Familial and Sporadic Pituitary Adenomas. Am J Hum Genet. 2017 May;100(5):817–23.

28. Hernández-Ramírez LC, Gam R, Valdés N, Lodish MB, Pankratz N, Balsalobre A, et al. Loss-of-function mutations in the CABLES1 gene are a novel cause of Cushing’s disease. Endocr Relat Cancer. 2017;24(8):379–92.

29. Hernández-Ramírez LC, Tatsi C, Lodish MB, Faucz FR, Pankratz N, Chittiboina P, et al. Corticotropinoma as a Component of Carney Complex. J Endocr Soc. 2017;1(7):918–25.

30. Radian S, Diekmann Y, Gabrovska P, Holland B, Bradley L, Wallace H, et al. Increased Population Risk of AIP-Related Acromegaly and Gigantism in Ireland. Hum Mutat. 2017;38(1):78–85.

31. Hernandez-Ramirez LC, Gabrovska P, Denes J, Stals K, Trivellin G, Tilley D, et al. Landscape of familial isolated and young-onset pituitary adenomas: Prospective diagnosis in AIP mutation carriers. J Clin Endocrinol Metab. 2015 Sep;100(9):E1242–54.

32. Iivonen A-P, Känsäkoski J, Karppinen A, Kivipelto L, Schalin-Jäntti C, Karhu A, et al. Screening for germline KCNQ1 and KCNE2 mutations in a set of somatotropinoma patients. Endocr Connect. 2018;7(5):645–52.

33. Caimari F, Hernández-Ramírez LC, Dang MN, Gabrovska P, Iacovazzo D, Stals K, et al. Risk category system to identify pituitary adenoma patients with AIP mutations. J Med Genet. 2018;55(4):254–60.

34. Nagata Y, Inoshita N, Fukuhara N, Yamaguchi-Okada M, Nishioka H, Iwata T, et al. Growth hormone-producing pituitary adenomas in childhood and young adulthood: clinical features and outcomes. Pituitary. 2018 Feb;21(1):1–9.

35. Makri A, Bonella MB, Keil MF, Hernandez-Ramirez L, Paluch G, Tirosh A, et al. Children with MEN1 gene mutations may present first (and at a young age) with Cushing disease. Clin Endocrinol (Oxf). 2018;89(4):437–43.

36. Scheithauer BW, Laws ERJ, Kovacs K, Horvath E, Randall R V, Carney JA. Pituitary adenomas of the multiple endocrine neoplasia type I syndrome. Semin Diagn Pathol. 1987 Aug;4(3):205–11.

37. Burgess JR, Shepherd JJ, Parameswaran V, Hoffman L, Greenaway TM. Prolactinomas in a large kindred with multiple endocrine neoplasia type 1: Clinical features and inheritance pattern. J Clin Endocrinol Metab. 1996;81(5):1841–5.

38. Burgess JR, Shepherd JJ, Parameswaran V, Hoffman L, Greenaway TM. Spectrum of pituitary disease in multiple endocrine neoplasia type 1 (MEN 1): Clinical, biochemical, and radiological features of pituitary disease in a large MEN 1 kindred. J Clin Endocrinol Metab. 1996;81(7):2642–6.

39. Vierimaa O, Georgitsi M, Lehtonen R, Vahteristo P, Kokko A, Raitila A, et al. Pituitary adenoma predisposition caused by germline mutations in the AIP gene. Science (80- ). 2006 May;312(5777):1228–30.

40. Daly AF, Vanbellinghen J-F, Khoo SK, Jaffrain-Rea M-L, Naves LA, Guitelman MA, et al. Aryl hydrocarbon receptor-interacting protein gene mutations in familial isolated pituitary adenomas: Analysis in 73 families. J Clin Endocrinol Metab. 2007 May;92(5):1891–6.

41. Cazabat L, Libè R, Perlemoine K, René-Corail F, Burnichon N, Gimenez-Roqueplo A-P, et al. Germline inactivating mutations of the aryl hydrocarbon receptor-interacting protein gene in a large cohort of sporadic acromegaly: Mutations are found in a subset of young patients with macroadenomas. Eur J Endocrinol. 2007 Jul;157(1):1–8.

42. Trouillas J, Labat-Moleur F, Sturm N, Kujas M, Heymann M-F, Figarella-Branger D, et al. Pituitary tumors and hyperplasia in Multiple Endocrine Neoplasia type 1 syndrome (MEN1): A case-control study in a series of 77 patients versus 2509 non-MEN1 patients. Am J Surg Pathol. 2008 Apr;32(4):534–43.

43. Igreja S, Chahal HS, King P, Bolger GB, Srirangalingam U, Guasti L, et al. Characterization of aryl hydrocarbon receptor interacting protein (AIP) mutations in familial isolated pituitary adenoma families. Hum Mutat. 2010 Aug;31(8):950–60.

44. Cain JW, Miljic D, Popovic V, Korbonits M. Role of the aryl hydrocarbon receptor-interacting protein in familial isolated pituitary adenoma. Expert Rev Endocrinol Metab. 2010;5(5):681–95.

45. de Pinho LKJ, Vieira Neto L, Wildemberg LEA, Moraes AB, Takiya CM, Frohman LA, et al. Familial isolated pituitary adenomas experience at a single center: clinical importance of AIP mutation screening. Arq Bras Endocrinol Metabol. 2010 Nov;54(8):698–704.

46. Vroonen L, Jaffrain-Rea M-L, Petrossians P, Tamagno G, Chanson P, Vilar L, et al. Prolactinomas resistant to standard doses of cabergoline: a multicenter study of 92 patients. Eur J Endocrinolgy. 2012 Nov;167(5):651–62.

47. Simonds WF, Varghese S, Marx SJ, Nieman LK. Cushing’s syndrome in multiple endocrine neoplasia type 1. Clin Endocrinol (Oxf). 2012 Mar;76(3):379–86.

48. Oriola J, Lucas T, Halperin I, Mora M, Perales MJ, Alvarez-Escolá C, et al. Germline mutations of AIP gene in somatotropinomas resistant to somatostatin analogues. Eur J Endocrinol. 2013 Jan;168(1):9–13.

49. Cuny T, Pertuit M, Sahnoun-Fathallah M, Daly A, Occhi G, Odou MF, et al. Genetic analysis in young patients with sporadic pituitary macroadenomas: Besides AIP don’t forget MEN1 genetic analysis. Eur J Endocrinol. 2013 Apr;168(4):533–41.

50. Preda V, Korbonits M, Cudlip S, Karavitaki N, Grossman AB. Low rate of germline AIP mutations in patients with apparently sporadic pituitary adenomas before the age of 40: A single-centre adult cohort. Eur J Endocrinol. 2014 Nov;171(5):659–66.

51. Schöfl C, Honegger J, Droste M, Grussendorf M, Finke R, Plöckinger U, et al. Frequency of AIP gene mutations in young patients with acromegaly: A registry-based study. J Clin Endocrinol Metab. 2014;99(12):E2789–93.

52. Mangupli R, Rostomyan L, Castermans E, Caberg J-H, Camperos P, Krivoy J, et al. Combined treatment with octreotide LAR and pegvisomant in patients with pituitary gigantism: clinical evaluation and genetic screening. Pituitary. 2016 Oct;19(5):507–14.

53. Matsumoto R, Izawa M, Fukuoka H, Iguchi G, Odake Y, Yoshida K, et al. Genetic and clinical characteristics of Japanese patients with sporadic somatotropinoma. Endocr J. 2016 Nov;63(11):953–63.

54. De Sousa SMC, McCabe MJ, Wu K, Roscioli T, Gayevskiy V, Brook K, et al. Germline variants in familial pituitary tumour syndrome genes are common in young patients and families with additional endocrine tumours. Eur J Endocrinol. 2017 May;176(5):635–44.

55. Ozkaya HM, Comunoglu N, Sayitoglu M, Keskin FE, Firtina S, Khodzhaev K, et al. Germline mutations of aryl hydrocarbon receptor-interacting protein (AIP) gene and somatostatin receptor 1–5 and AIP immunostaining in patients with sporadic acromegaly with poor versus good response to somatostatin analogues. Pituitary. 2018;21(4):335–46.

56. Marques P, Palou FC, Hernández-Ramírez LC, Barry S, Iacovazzo D, Grossman A, et al. Significant phenotypic difference between clinically presenting vs prospectively diagnosed pituitary adenoma in AIP mutation-positive kindreds. In: 100th Annual Meeting of the Endocrine Society, ENDO 2018. P. Marques, Centre for Endocrinology, William Harvey Research Institute, Queen Mary University of London, London, United Kingdom; 2018. p. Mar 17-20, Chicago, IL, United States.
