# Supplementary material for: Clinical Relevance of Genetic Analysis in Patients With Pituitary Adenomas: A Systematic Review
Source: Front Endocrinol (Lausanne). 2019 Dec 10;10:837. doi: 10.3389/fendo.2019.00837 (PMC6914701; doi:10.3389/fendo.2019.00837)
Supplement: Supplementary file 3 [file Data_Sheet_3.docx]

**Supplemental Material 3: Format of Quality Assessment: Adjusted Quality Assessment of Diagnostic Accuracy Studies tool (QUADAS-2)**

**Risk of bias**

1A1: Was a consecutive or random sample of patients with sporadic pituitary tumours enrolled?

In order to determine if enrollment took place in a consecutive or random manner – taking into account the applied in/exclusion criteria –, it was necessarily to be informed about the inclusion place(s) and inclusion period.

If patient selection took place by enrollment of patients in a consecutive or random manner, the study is scored “yes”.

If patient selection did not take place by enrollment of patients in a consecutive or random manner, or if this could not be determined due to a lack of information, the study is scored “no”.

1A2: Did the study avoid inappropriate exclusions?

Exclusion of patients based on familial pituitary tumors is scored as “yes” (= appropriate exclusion)

Exclusion of sporadic patients with syndromic features suspected for a specific germline mutation is scored as “yes” (= appropriate exclusion).

Exclusion of patients with a proven germline mutation (e.g. exclusion of MEN1 patients when *AIP* gene mutation is investigated) is scored as “yes” (= appropriate exclusion).

Including only a subgroup of patients with sporadic pituitary tumours based on potential predictors of germline mutations such as age, hormonal overproduction, tumor size or patient length is scored as “yes” (appropriate)

Exclusion of patients in which certain germline mutations are already investigated but proved to be non-carriers (e.g. exclusion of patients in which *MEN1* mutation carriership was investigated earlier, but showed no pathogenic mutations, when *AIP* gene mutation is investigated) is scored as “no” (= inappropriate exclusion).

In/excluding a subgroup of patients based on other (additional) criteria is scored as “no”.

If no (additional) exclusion criteria are used or described, the study is scored “yes”.

1A3: Was the patient sample population based?

If the study population was based on a population based database/network, it is scored “yes”.

If not (or unclear), it is scored “no”.

**1A:**

**If all 3 questions are answered “yes”, the study is scored + + on patient selection**

**If 2 questions are answered “yes”, the study is scored + on patient selection**

**If 1 question is answered “yes”, the study is scored - on patient selection**

**If no question is answered “yes”, the study is scored - - on patient selection**

3A1: Is the reference standard likely to correctly classify the target condition: is the proper method used to find DNA sequence variations?

Coding exome sequencing is classified as adequate reference standard. In case of investigating *Xq26.3* microduplications, Copy Number Variation (CNV) analysis or comparative genomic hybridization microarray (aCGH) must be used.

If this is performed correctly, the study is scored “yes”.

If not, it is scored “no”. SSCP (single strand conformation polymorphism ) alone is not considered an appropriate method for sequencing, and scored “no”.

3A2: Is the reference standard likely to correctly classify the target condition: is the proper method used to find DNA copy number variations (CNV)?

Studies investigating germline mutations and/or CNVs in *AIP*, *MEN1* and/ or *CDKN1B* gene, multiplex ligation-dependent probe amplification (MLPA) must be used in addition to coding exome sequencing as adequate reference standard.

If this method is used when investigating the *AIP*, *MEN1* and/or *CDKN1B* gene, the study is scored “yes”.

If not, it is scored “no”.

3A3: Is the reference standard likely to correctly classify the target condition: is the proper method used to investigate the interpretation of found mutations?

The pathogenicity of a genetic variation should have been investigated with more than one of the following tools: investigating the frequency of variation in healthy controls, investigating the frequency of variations in reference databases, in silico analysis, functional studies and/or evidence on pathogenicity reported in literature.

If pathogenicity is investigation more than one mentioned methods, the study is scored “yes”.

If not, it is scored “no”

**3A:**

**If all 3 questions are answered “yes”, the study is scored + + on reference standard**

**If 2 questions are answered “yes”, the study is scored + on reference standard**

**If 1 question is answered “yes”, the study is scored - on reference standard**

**If no question is answered “yes”, the study is scored - - on reference standard**

**NB: when question 3A2 is not applicable (a study is not investigating AIP, MEN1 or CDKN1B), a study is scored + + when both questions are answered “yes”. If 1 question is answered “yes, the study is scored + -. If no question is answered “yes”, it is scored - -**

4A1: Did all patients receive a reference standard?

It should be clear that all patients received a reference standard. In that case, a study is scored “yes”.

If not (e.g. due to no informed consent for genetic analysis), a study is scored “no”.

4A3: Did all patients receive the same reference standard?

A low risk of biased was given when all patients underwent the same genetic analysis. The study is scored “yes”.

In all other cases, it is scored “no”.

4A4: Were all patients included in the analysis?

All patients should be included in the analysis. In that case, a study is scored “yes”.

If not, it is scored “no”.

**4A:**

**If all 3 questions are answered “yes”, the study is scored + + on flow and timing**

**If 2 questions are answered “yes”, the study is scored + - on flow and timing**

**If 1 question is answered “yes”, the study is scored - on flow and timing**

**If no question is answered “yes”, the study is scored - - on flow and timing**

**Applicability**

1B: Are there concerns that the included patients do not match the review question?

This review focusses on patients with sporadic pituitary adenoma without syndromic features suggestive for a (certain) germline mutation due to the presence of additional endocrine tumors in personal or family history. This should be the study domain of the included study. In that case, the study is scored “+”.

If additional in/exclusion criteria are used (e.g. age criteria, hormonal tumor production, tumor size, patient length), it introduces a difference between the study population and the target population, and the study is scored “+/-“.

If there are not enough baseline data reported to make a fair judgement about the applicability, the study is scored “-“.

The minimum required data are: gender, age (of diagnosis or start of symptoms), familial status, subtype tumor.

3B: Are there concerns that the target condition as defined by the reference standard does not match the review question?

The presence of a germline mutation must have been evaluated using whole exome sequencing and – in the case of *AIP*, *MEN1* or *CDKN1B* – also by MLPA. If this is the case, the study is scored “+”.

In case of investigation of *AIP*, *MEN1* or *CDKN1B* gene: if MLPA is not used, the study is scored as “+/-“.

The pathogenicity must have been investigated, only pathogenic and likely pathogenic mutations using the classification of the Association for Clinical Genetics Science (ACGS) and American College of Medical Genetics and Genomics (ACMG) guidelines are considered as clinically relevant.(1,2) If a study does not evaluate mutations in terms of pathogenicity, the study is scored as “-“.

If a study does not investigate the entire coding exome by (direct) sequencing (or in case of *Xq26.3* microduplication: aCGH or CNV analysis), the study is scored as “-“.

**References**

1. Wallis Y, Payne S, McAnulty C, Bodmer D, Sistermans E, Robertson K, Moore D, Abbs S, Deans Z & Devereau A. Practice guidelines for the evaluation of pathogenicity and the reporting of sequence variants in clinical molecular genetics. *Association for Clinical Genetic Science and the Dutch Society of Clinical Genetic Laboratory Specialists*, 2013.

2. Richards S, Aziz N, Bale S, Bick D, Das S, Gastier-Foster J, Grody WW, Hegde M, Lyon E, Spector E *et al*. Standards and guidelines for the interpretation of sequence variants: a joint consensus recommendation of the American College of Medical Genetics and Genomics and the Association for Molecular Pathology. *Genetics in Medicine* 2015 17 405–424.
