# Supplementary material for: Clinical Relevance of Genetic Analysis in Patients With Pituitary Adenomas: A Systematic Review
Source: Front Endocrinol (Lausanne). 2019 Dec 10;10:837. doi: 10.3389/fendo.2019.00837 (PMC6914701; doi:10.3389/fendo.2019.00837)
Supplement: Supplementary file 4 [file Data_Sheet_4.docx]

**Supplemental Material 4: Format of Quality Assessment: Adjusted Quality In** Prognosis Studies (QUIPS)

**1. Study participation and attrition**

a. Study population is adequately described

Yes: Baseline table or adequate description must be present (gender, age, sporadic/familial status, type of adenoma, tumor size (or macroadenoma yes/no), any other endocrine tumours and/or syndromic features (if applicable), genetic status (if already partly investigated).

No: no adequate description or only some characteristics addressed.

b. Inclusion and exclusion criteria are adequately described

Yes: the inclusion and exclusion criteria are adequately described, including recruitment period and place(s).

No: insufficient description.

c The data from an adequate proportion of the study population are available for outcome measurement

Yes: Number of patients lost to follow-up and the reasons for loss to follow-up and drop out are adequately described.

No: Insufficient description of participation in outcome measurement (treatment outcome) or if there is reason to believe that the selection of patients who lost to follow-up differs from the rest of the study population.

d. The study population is population based

If the study population was based on a population based database/network, it is scored “yes”.

If not (or unclear), it is scored “no”.

**If all 4 questions are answered “yes”, the study is scored + + on patient selection**

**If 3 questions are answered “yes”, the study is scored + on patient selection**

**If 2 questions are answered “yes”, the study is scored + - on patient selection**

**If 1 question is answered “yes”, the study is scored - on patient selection**

**If no question is answered “yes”, the study is scored - - on patient selection**

**2. Prognostic factor measurement (germline mutation)**

a. a valid and reliable modality for investigating germline status was used

Yes: the combination of coding exome sequencing and – in case of investigation of germline mutations in *MEN1*, *AIP* or *CDKN1B* – multiplex ligation-dependent probe amplification was used. In case of investigating *Xq26.3* microduplications, Copy Number Variation (CNV) analysis or comparative genomic hybridization microarray (aCGH) must be used. Furthermore, more than one method must have been used to investigate the pathogenicity of a genetic variation (frequency of variation in healthy controls, frequency of variations in reference databases, in silico analysis, functional studies and/or evidence on pathogenicity reported in literature)

No: inadequate determination of germline status and/or investigation on pathogenicity of DNA variants

b. adequate participation

Yes: All study participants have been investigated for germline mutations.

No: not all study participants have been investigated for germline mutations.

**If all 2 questions are answered “yes”, the study is scored + + on prognostic factor measurement**

**If 1 question is answered “yes”, the study is scored + - on prognostic factor measurement**

**If no question is answered “yes”, the study is scored - - on prognostic factor measurement**

**3. Outcome measurement (treatment outcome)**

a. The investigated outcome is predefined and a clear definition of outcome is provided (including duration and follow-up)

Yes: adequate description of investigated outcome (predefined). In case of follow-up: duration of follow-up and explicit description of investigations performed during that period must be described.

No: no sufficient description of outcome definition(s), follow-up measurements and/or duration.

b. The method of outcome measurement used is adequately valid and reliable

Yes: Method of outcome measurement (treatment characteristics/outcome) must be described properly. In case of follow-up :this must be adequate to assess clinical relevant variations.

No: no adequate outcome measurement.

c. The method and setting for measurement is the same for all participants

Yes: outcome measurements are the same for patients with and without germline mutation.

No: Relevant difference in outcome measurements between patients with germline mutation vs non-carriers.

**If all 3 questions are answered “yes”, the study is scored + + on outcome measurement**

**If 2 questions are answered “yes”, the study is scored + - on outcome measurement**

**If 1 question is answered “yes”, the study is scored - on outcome measurement**

**If no question is answered “yes”, the study is scored - - on outcome measurement**

**4. Analysis and reporting**

a. Sufficient presentation of data to assess the relationship between prognostic factor and outcome

Yes: sufficient description of data to assess the relationship between germline mutation and treatment outcome.

No: insufficient description.

b. There is no selective reporting of results

Yes: no selective reporting

No: selective reporting

**If all 2 questions are answered “yes”, the study is scored + + on analysis and reporting**

**If 1 question is answered “yes”, the study is scored + - on outcome measurement**

**If no question is answered “yes”, the study is scored - - on outcome measurement**
