# Supplementary material for: Clinical Relevance of Genetic Analysis in Patients With Pituitary Adenomas: A Systematic Review
Source: Front Endocrinol (Lausanne). 2019 Dec 10;10:837. doi: 10.3389/fendo.2019.00837 (PMC6914701; doi:10.3389/fendo.2019.00837)
Supplement: Supplementary file 5 [file Data_Sheet_5.docx]

**Supplemental Material 5 Study results**

***Table 1: Predictors on germline mutation status in sporadic PA***

**1A. Sporadic somatotroph adenoma**

| **Author & year** | **Investigated gene** | **Predictor** | **Comparison of groups** | **Outcome** | **Statistical significance** |
| --- | --- | --- | --- | --- | --- |
| Cazabat  2007 (1) | *AIP* | Younger age | Mean age ± SD of *AIP* mutated patients  vs mean ± SD age of wildtype | 25 ± 10 years vs  43 ± 14 years | P=0.005 |
|  |  | Gigantism | Frequency of gigantism in *AIP* mutated patients  vs wildtype | 3/5 (60%) vs  17/149 (11.4%) | P=0.016 |
|  |  | Male gender | Frequency of male gender in *AIP* mutated patients  vs wildtype | 4/5 (80%) vs  66/149 (44.3%) | P=0.12 |
| Trivellin  2014 (2) | *X26.3* microduplication^a^ | Female gender | Frequency of female gender in patients with *Xq26.3* microduplication vs wildtype | 10/14 (71%) vs  7/29 (42%) | P=0.007 |
|  |  | Onset of rapid growth | Median age (and range) at onset of rapid growth in patients with *Xq26.3* microduplication vs wildtype | 1.0 year (0.5 – 2.0) vs  16.0 year (range 5.0 – 18.0) | P<0.001 |
|  |  | IGF-1 level at diagnosis | Median factor increase (multiple of ULN) (and range) of IGF-1 at diagnosis in patients with *Xq26.3* microduplication vs wildtype | 4.4 (range 2.4 – 5.2) vs  2.1 (range 1.4 – 5.3) | P=0.005 |
|  |  | Elevated prolactin levels at diagnosis | Number of patients with elevated prolactin levels at diagnosis in patients with *Xq26.3* microduplication vs wildtype | 13/14 (93%) vs  6/29 (21%) | P<0.001 |

**Legend table 1A**

IGF-1: insulin-like growth factor 1, ULN: upper limit of normal.

a: these results included 9 sporadic and 5 familial cases of *Xq26.3* microduplication.

*Young (≤30 years) patients with sporadic pituitary adenoma*

**1B. Young (≤30 years) sporadic adenoma**

| **Author & year** | **Investigated gene** | **Predictor** | **Comparison of groups** | **Outcome** | **Statistical significance** |
| --- | --- | --- | --- | --- | --- |
| Tichomirowa 2011 (3) | *AIP* | Younger age | Prevalence of *AIP* mutation < 18 years vs  ≥ 18 years at diagnosis | 8/39 (20.5%) vs  11/124 (8.9%) | P<0.01 |
|  |  | Macroadenoma | N/A |  |  |
|  |  | Gigantism | N/A |  |  |
|  |  | Male gender | Frequency of males in *AIP* mutated patient vs wildtype | 16/19 (84.2%) vs  68/144 (47.2%) | Not reported |
|  |  | Adenoma subtype | Prevalence of *AIP* mutation in somatotroph adenomas vs prolactinomas vs NFPA | 11/83 (13.3%) vs  7/61 (11.5%) vs  1/16 (6.3%) | Not reported |
|  |  | Extrasellar extension | Frequency of extrasellar extention in *AIP* mutated patients | 14/17^a^ (82.4%) | N/A |
| Cuny 2013 (4) | *AIP* | Younger age | Prevalence of *AIP* mutation ≤ 18 years vs  > 18 years at diagnosis | 7/46 (15.2%) vs  8/128 (6.3%) | Not reported |
|  |  | Macroadenoma | N/A |  |  |
|  |  | Gigantism | Prevalence of *AIP* mutation in giants vs  non-giants | 3/6 (50%) vs  12/168 (7.1%) | Not reported |
|  |  | Male gender | Frequency of males in *AIP* mutated patient vs wildtype | 11/15 (73.3) vs  85/159 (53.5%) | Not reported |
|  |  | Adenoma subtype | Prevalence of *AIP* mutation in NFPA vs  non-NFPA | 3/12 (25%) vs  12/162 (7.4%) | Not reported |
|  |  | Extrasellar extension | Frequency of extrasellar extention in *AIP* mutated patients | 15/15 (100%) | N/A |
|  | *MEN1* | Younger age | Prevalence of *MEN1* mutation ≤ 18 years vs  > 18 years at diagnosis | 3/46 (6.5%) vs  3/128 (2.3%) | Not reported |
|  |  | Adenoma subtype | Prevalence of *MEN1* mutation in prolactinoma vs  non-prolactinoma | 4/74 (5.4%) vs  2/100 (2%) | Not reported |
| Hernandez 2015 (5) | *AIP* | Younger age | Median age (and IQR) at onset in *AIP* mutated patients vs wildtype | 16 years (14.8 – 22.3) vs  22 years (16 – 26) | P=0.0054 |
|  |  |  | Percentage of pediatric patients in *AIP*  mutated patients vs wildtype | 58.8% vs  35.9% | P=0.0085 |
|  |  | Macroadenoma | Frequency of macroadenoma in *AIP* mutated patient vs wildtype | 29/29 (100%) vs  283/328 (86.3%) | Not reported |
|  |  |  | Frequency of adenomas *≥40* mm in *AIP* mutated patient vs wildtype | Not reported | P=0.7859 |
|  |  |  | Maximum diameter in *AIP* mutated patient vs wildtype | Not reported | P=0.6965 |
|  |  | Gigantism | Prevalence of *AIP* mutation in sporadic patients with gigantism (no comparison) | 19/75 (25.3%) |  |
|  |  | Male gender | Frequency of males in *AIP* mutated patients vs wildtype | 61.8% vs  49.2% | P=0.1605 |
|  |  | Adenoma subtype^b^ | Frequency of somatotroph adenoma in *AIP* mutated patients vs wildtype | 100% vs  69.2% | Not reported |
|  |  | Extrasellar extension | Frequency of extrasellar extention in *AIP* mutated patients vs wildtype | 95% vs  58.9% | P=0.0011 |

**Legend table 1B**

IQR: interquartile range. N/A: not applicable. NFPA: non-functioning pituitary adenoma

a: 17 out of 19 tumours from *AIP* mutated patients were investigated on extrasellar extension
b: proven by clinical diagnosis and immunohistochemistry

**1C. Other groups of patients with sporadic pituitary adenoma**

| **Author & year** | **Investigated gene** | **Predictor** | **Comparison of groups** | **Outcome** | **Statistical significance** |
| --- | --- | --- | --- | --- | --- |
| Cai  2013 (6) | *AIP* | Younger age | Prevalence of *AIP* mutation ≤ 18 years vs  > 18 years at diagnosis | 1/11 (9.1%) vs  5/205 (2.4%) | Not reported |
|  |  | Adenoma subtype | Prevalence of *AIP* mutation in somatotroph adenoma vs non-somatotroph adenoma | 5/80 (6.3%) vs  1/136 (0.7%) | Not reported |
|  |  | Male gender | Prevalence of *AIP* mutation in males vs females | 5/94 (5.3%) vs  1/122 (0.8%) | Not reported |

***Table 2: Impact of a germline mutation on treatment outcome in pituitary adenoma***

| **Author & year** | **Investigated gene** | **Comparison of groups** | **Treatment (outcome)** | **Results** | **Statistical significance** |
| --- | --- | --- | --- | --- | --- |
| Daly  2010 (7) | *AIP* | *AIP* mutated vs wildtype somatotroph adenomas | Proportion multimodal treatment  (≥2 modalities) | 61.3% vs 66.4% | Not reported |
|  |  |  | Proportion neurosurgery | 87.3% vs 80.5% | Not reported |
|  |  |  | Proportion reoperation | 21.9% vs 5.5% | P=0.00069 |
|  |  |  | Proportion radiotherapy | 41.4% *vs* 24.7% | P=0.15 |
|  |  |  | Long-term (≥12 months) disease control | 50/71 (70.4%) vs 182/226 (80.5%) | P=0.06 |
|  |  |  | Median SSA-induced GH reduction | 40% (range 0-99%)^a^ vs  75% (range 0-99%)^b^ | P=0.0004 |
|  |  |  | Median SSA-induced IGF-1 reduction | 47.4% (range 0-83.4%)^a^ vs 56.0% (range: 0-100%)^b^ | P=0.028 |
|  |  |  | Median SSA-induced tumor shrinkage | 0% (range 0-90%)^a^ vs 41.4% (range 0-95%)^b^ | P<0.000001 |
|  |  |  | Disease control rate achieved with  SSA as primary treatment | 1/6 vs 17/32 | Not reported |
|  |  |  | Disease control rate achieved with  SSA as preoperative treatment | 1/6 vs 6/16 | Not reported |
|  |  |  | Disease control rate achieved with  SSA as postoperative treatment | 9/26 vs 51/84 | Not reported |
|  |  |  | Proportion disease control with pegvisomant | 1/4 vs 19/19 | Not reported |
|  |  |  | Frequency of hypopituitarism | 22.5% vs 25.2% | Not reported |
|  |  |  | Number of deficient pituitary hormonal axes | Not reported | P<0.000001 |
|  |  | *AIP* mutated vs wildtype somatotroph adenomas with high cumulative treatment burden (≥3 modalities) | Long-term (≥12 months) disease control | 15/27 (55.6%) vs  63/76 (82.9%) | P=0.01 |
| Nagata  2018 (8) |  | *AIP* mutated vs wildtype somatotroph adenomas | Hormonal control at time of last follow-up | (3/5) 60% vs  (16/18) 88.9% | Not reported |
|  |  |  | Remission by surgery alone | (2/5) 20% vs (12/18) 66.7% | Not reported |
| Tichomirowa 2011 (3) |  | *AIP* mutated somatotroph adenomas (n=11) | Proportion ≥ two surgical interventions | 4/11 (36.4) | N/A |
|  |  |  | Disease control by secondary SSA therapy | 1/9 (11.1%) | N/A |
|  |  |  | Tumoursize reduction by SSA treatment | 1/6 (16.7%) | N/A |
| Rostomyan  2015 (9) |  | *AIP* mutated vs wildtype pituitary giants | GH/IGF-1 control at last follow-up | 61.0% vs 43.0% | P=0.03 |
|  |  |  | GH/IGF-1 control < 19 years | 72.7% vs 22.7% | P=0.0001 |
|  |  |  | GH/IGF-1 control before final height | 48.6% vs 10.9% | P<0.0001 |
|  |  |  | Median age when first control achieved | 17.3 (IQR 15 – 20) vs  27 (IQR 18 – 37) | P<0.0001 |
|  |  |  | Proportion multimodal treatment (≥3 modalities) | 23.8% vs 42.7% | P=0.04 |
|  |  |  | Long-term control (>12 months) | 55.3% vs 38.4% | P=0.08 |
|  |  |  | Frequency of hypopituitarism | 73% vs 66% | P=0.4 |
| Iacovazzo 2016 (10) |  | *AIP* mutated vs wildtype pituitary giants | Median number of treatments | 2 (range 1-4) vs  3 (range 1-4) | Not significant |
|  |  |  | Frequency of hypopituitarism | 13/28 (46.4%) vs  11/19 (57.9%) | Not significant |
| Salenave  2015 (11) |  | DA-resistant adenoma vs  DA-sensitive adenoma | Prevalence of *AIP* mutation | 2/17 (11%) vs  2/37% (5%) | Not significant |
| Tichomirowa 2011 (3) |  | *AIP* mutated prolactinomas (n=7) | Disease control by DA treatment | 3/6 (50%) | N/A |
|  |  |  | Proportion neurosurgery | 5/7 (71.4%) | N/A |
|  |  |  | Proportion multiple surgeries | 4/7 (66.7%) | N/A |
| Daly  2010 (7) |  | *AIP* mutated prolactinomas (n=13) | Disease control by DA treatment | 6/12 (50%) | N/A |
|  |  |  | Proportion neurosurgery | 7/13 (53.8%) | N/A |
|  |  |  | Proportion multiple surgeries | 4/13 (30.8%) | N/A |
|  |  |  | Long-term hormonal control | 8/13 (61.5%) | N/A |
| Daly  2010 (7) |  | *AIP* mutated NFPA (n=7) | Proportion neurosurgery | 6/7 (85.7%) | N/A |
|  |  |  | Long-term control of tumour size | 7/7 (100%) | N/A |
| Caimari  2018 (12) |  | *AIP* mutated PA vs wildtype | Median number of treatments | 2 (IQR 1-3) vs  1 (IQR 1-2) | P=0.055 |
| De Laat  2015 (13) | *MEN1* | *MEN1* mutated prolactinomas | Hormonal control by DA  As primary treatment | 27/30 (90.0%) | N/A |
|  |  |  | Hormonal control by DA  as secondary treatment | 8/9 (88.9%) | N/A |
|  |  |  | Proportion neurosurgery | 4/52 (7.7%) | N/A |
|  |  | *MEN1* mutated NPFA | Proportion neurosurgery | 8/52 (15.4%) | N/A |
|  |  |  | Proportion stable tumour size in initially untreated cases | 40/45 (88.9%) | N/A |
| Verges  2002 (14) |  | *MEN1* mutated vs wildtype functional PA | Proportion normalization of pituitary hypersecretion | 49/116 (42%) vs 83/110 (90%) | P<0.001 |
|  |  | *MEN1* mutated vs wildtype prolactinoma | Proportion of normalization of plasma prolactin | 37/85 (44%) vs (90%) | P<0.001 |
| Salenave  2015 (11) |  | DA-resistant adenoma vs  DA-sensitive adenoma | Prevalence of *MEN1* mutation | 3/18 (16%) vs  0/40% (0%) | P=0.026^c^ |
| Rostomyan  2015 (9) | *Xq26.3* microduplication | Pituitary giants with *Xq26.3* microduplication vs wildtype pituitary giants | GH/IGF-1 control at last follow-up | 58.0% vs 43.0% | P=0.02 |
|  |  |  | GH/IGF-1 control < 19 years | 85.7% vs 22.7% | Not reported |
|  |  |  | GH/IGF-1 control before final height | 54.5% vs 10.9% | P<0.0001 |
|  |  |  | Median age when first control achieved | 8.0 (IQR 4-13) vs  27 (IQR 18-37) | P=0.0005 |
|  |  |  | Proportion multimodal treatment (≥3 modalities) | 46.0% vs 42.7% | P=0.6 |
|  |  |  | Long-term control (>12 months) | 41.7% vs 38.4% | P=0.1 |
|  |  |  | Frequency of hypopituitarism | 75% vs 66% | P=0.7 |
| Iacovazzo  2016 (10) |  | Pituitary giants with *Xq26.3* microduplication vs wildtype pituitary giants | Median number of treatments | 3.5 (IQR 2 – 4.7) vs  3 (IQR 1 – 4) | Not significant |
|  |  |  | Frequency of hypopituitarism | 8/12 (66.7%) vs  11/19 (57.9%) | Not significant |
|  |  | Pituitary giants with *Xq26.3* microduplication (n=12) | Disease control at last follow-up | 11/12 (91.7%) | N/A |
|  |  |  | Proportion multimodal ((≥2) treatments | 9/12 (75%) | N/A |
|  |  |  | Frequency of hypopituitarism | 8/12 (66.7%) | N/A |
| Beckers  2015 (15) |  | Pituitary giants with *Xq26.3* microduplication (n=18) | Hormonal control at last follow-up | 14/18 (77.8%) | N/A |
|  |  |  | Control of excessive growth | 13/17 (76.5%) | N/A |
|  |  |  | Proportion multimodal ((≥2) treatments | 14/18 (77.8%) | N/A |
|  |  |  | Proportion neurosurgery | 17/18 (94.4%) | N/A |
|  |  |  | Frequency of hypopituitarism | 12/17 (70.6%) | N/A |
|  |  |  | Hormonal control by DA or SSA as primary treatment | 0/9 (0%) | N/A |
|  |  |  | Median reduction of GH/IGF-1 by SSA as primary treatment | 37.5% | N/A |
|  |  |  | Median reduction of GH/IGF-1 by SSA as secondary treatment | 14.2% | N/A |
|  |  |  | Hormonal control by surgery as primary treatment | 3/9 (33.3%) | N/A |

**Legend table 2**

DA: dopamine agonist, GH: growth hormone, IGF-1: insulin-like growth factor 1, IQR: interquartile range, N/A: not applicable, NFPA: non functioning pituitary adenoma , SSA: somatostatin analogue.

a: number of *AIP* mutated patients with long-term SSA treatment: 38

b:number of controls with long-term SSA treatment: 164

c: regression analysis: the presence of a *MEN1* mutation showed to be a significant and independent predictor of dopamine agonist (t=3.052, P=0.004)

**References**

1. Cazabat L, Libè R, Perlemoine K, René-Corail F, Burnichon N, Gimenez-Roqueplo A-P, et al. Germline inactivating mutations of the aryl hydrocarbon receptor-interacting protein gene in a large cohort of sporadic acromegaly: Mutations are found in a subset of young patients with macroadenomas. Eur J Endocrinol. 2007 Jul;157(1):1–8.

2. Trivellin G, Daly AF, Faucz FR, Yuan B, Rostomyan L, Larco DO, et al. Gigantism and acromegaly due to Xq26 microduplications and GPR101 mutation. N Engl J Med. 2014 Dec;371(25):2363–74.

3. Tichomirowa MA, Barlier A, Daly AF, Jaffrain-Rea ML, Ronchi C, Yaneva M, et al. High prevalence of AIP gene mutations following focused screening in young patients with sporadic pituitary macroadenomas. Eur J Endocrinol. 2011;165(4):509–15.

4. Cuny T, Pertuit M, Sahnoun-Fathallah M, Daly A, Occhi G, Odou MF, et al. Genetic analysis in young patients with sporadic pituitary macroadenomas: Besides AIP don’t forget MEN1 genetic analysis. Eur J Endocrinol. 2013 Apr;168(4):533–41.

5. Hernandez-Ramirez LC, Gabrovska P, Denes J, Stals K, Trivellin G, Tilley D, et al. Landscape of familial isolated and young-onset pituitary adenomas: Prospective diagnosis in AIP mutation carriers. J Clin Endocrinol Metab. 2015 Sep;100(9):E1242–54.

6. Cai F, Zhang Y-D, Zhao X, Yang Y-K, Ma S-H, Dai C-X, et al. Screening for AIP gene mutations in a Han Chinese pituitary adenoma cohort followed by LOH analysis. Eur J Endocrinol. 2013 Dec;169(6):867–84.

7. Daly AF, Tichomirowa MA, Petrossians P, Heliovaara E, Jaffrain-Rea M-L, Barlier A, et al. Clinical characteristics and therapeutic responses in patients with germ-line AIP mutations and pituitary adenomas: An international collaborative study. J Clin Endocrinol Metab. 2010 Nov;95(11):E373–83.

8. Nagata Y, Inoshita N, Fukuhara N, Yamaguchi-Okada M, Nishioka H, Iwata T, et al. Growth hormone-producing pituitary adenomas in childhood and young adulthood: clinical features and outcomes. Pituitary. 2018 Feb;21(1):1–9.

9. Rostomyan L, Daly AF, Petrossians P, Nachev E, Lila AR, Lecoq A-L, et al. Clinical and genetic characterization of pituitary gigantism: An international collaborative study in 208 patients. Endocr Relat Cancer. 2015 Oct;22(5):745–57.

10. Iacovazzo D, Caswell R, Bunce B, Jose S, Yuan B, Hernández-Ramírez LC, et al. Germline or somatic GPR101 duplication leads to X-linked acrogigantism: a clinico-pathological and genetic study. Acta Neuropathol Commun. 2016 Jun;4(1):56.

11. Salenave S, Ancelle D, Bahougne T, Raverot G, Kamenický P, Bouligand J, et al. Macroprolactinomas in children and adolescents: Factors associated with the response to treatment in 77 patients. J Clin Endocrinol Metab. 2015;100(3):1177–86.

12. Caimari F, Hernández-Ramírez LC, Dang MN, Gabrovska P, Iacovazzo D, Stals K, et al. Risk category system to identify pituitary adenoma patients with AIP mutations. J Med Genet. 2018;55(4):254–60.

13. de Laat JM, Dekkers OM, Pieterman CRC, Kluijfhout WP, Hermus AR, Pereira AM, et al. Long-term natural course of pituitary tumors in patients with MEN1: Results from the dutchmen1 study group (DMSG). J Clin Endocrinol Metab. 2015 Sep;100(9):3288–96.

14. Vergès B, Boureille F, Goudet P, Murat A, Beckers A, Sassolas G, et al. Pituitary disease in MEN type 1 (MEN1): Data from the France-Belgium MEN1 multicenter study. J Clin Endocrinol Metab. 2002;87(2):457–65.

15. Beckers A, Lodish MB, Trivellin G, Rostomyan L, Lee M, Faucz FR, et al. X-linked acrogigantism syndrome: Clinical profile and therapeutic responses. Endocr Relat Cancer. 2015 Jun;22(3):353–67.
